# Investigating the neural and behavioral correlates of the stress-rumination link in healthy humans by modulating the left Dorsolateral Prefrontal Cortex using Theta Burst Stimulation

**DOI:** 10.3758/s13415-025-01305-0

**Published:** 2025-06-23

**Authors:** Isabell Int-Veen, Ute Eßer, Sandra Ladegast, Leonhard Liermann, Ramona Täglich, Betti Schopp, Hans-Christoph Nuerk, Christian Plewnia, Agnes Kroczek, Stefanie De Smet, Marie-Anne Vanderhasselt, Andreas J. Fallgatter, Ann-Christine Ehlis, Beatrix Barth, David Rosenbaum

**Affiliations:** 1 Tübingen Center for Mental Health (TüCMH), Department of Psychiatry and Psychotherapy, University Hospital Tübingen, Tübingen, Germany; 2 Department of Psychology, University of Tübingen, Tübingen, Germany; 3 German Center for Mental Health (DZPG), partner site Tübingen, Tübingen, Germany; 4 Department of Head and Skin, Faculty of Medicine and Health Sciences, Ghent University, Ghent, Belgium; 5 Ghent Experimental Psychiatry (GHEP) lab, Ghent, Belgium; 6 Brain Stimulation and Cognition (BSC) Lab, Department of Cognitive Neuroscience, Faculty of Psychology & Neuroscience, Maastricht University, Maastricht, The Netherlands; 7 LEAD Graduate School & Research Network, University of Tübingen, Tübingen, Germany

**Keywords:** Theta Burst Stimulation, Stress, Rumination, Trier Social Stress Test, Dorsolateral Prefrontal Cortex

## Abstract

**Abstract:**

Theta Burst Stimulation (TBS) offers a noninvasive way to modulate neural activation patterns, helping to explore the causal role of brain regions in psychiatric symptoms. Prefrontal hypoactivation is commonly observed in high ruminators and patients with depression during stress. However, the impact of modulating Dorsolateral Prefrontal Cortex (DLPFC) activity via excitatory and inhibitory TBS during stress remains unexplored. We studied 88 healthy participants (44 low, 44 high ruminators), each attending two appointments that included stress induction using the Trier Social Stress Test (TSST) and cortical oxygenation assessment with functional Near-Infrared Spectroscopy (fNIRS). Participants received either intermittent TBS (iTBS) or continuous TBS (cTBS) applied to the left DLPFC, with sessions randomized between active and sham stimulation. While TBS had no impact on positive affect, TSST performance, or heart rate, we observed effects on stress, state rumination, negative affect, and cortical oxygenation. We observed higher stress and higher negative affect during and after the TSST in high ruminators receiving iTBS compared with sham TBS (sTBS). Low ruminators showed reduced state rumination increases after iTBS compared with sTBS at their second appointment. fNIRS data revealed cortical oxygenation differences during the TSST, although only without multiple comparison corrections. Descriptively, we observed higher activation in the left Ventrolateral Prefrontal Cortex (VLPFC) following cTBS compared with sTBS in high ruminators but lower cortical oxygenation following cTBS compared with sTBS in low ruminators but only when participants received active stimulation first. This suggests stimulation sequence affects repeated-measures TMS studies in stress contexts. Findings highlight expectancy effects and suggest a potential reduction in TBS impact due to strong hemodynamic responses during stress.

**Highlights:**

• High ruminators showed increased stress and negative affect after iTBS during the TSST.

• Neural data showed increased cortical oxygenation in the left Ventrolateral Prefrontal Cortex (VLPFC) following cTBS to the left Dorsolateral Prefrontal Cortex (DLPFC) in high ruminators.

• In low ruminators, cTBS led to decreased activation, but only when active stimulation was administered first, highlighting the role of stimulation order in TBS outcomes.

• Expectancy effects and habituation are important aspects to be considered in repeated measures designs involving TBS and stress-reactive rumination.

**Supplementary Information:**

The online version contains supplementary material available at 10.3758/s13415-025-01305-0.

## Introduction

With Non-Invasive Brain Stimulation (NIBS), the investigation of brain functioning in mental disorders has gained a highly promising tool to evaluate the involvement of brain regions in altered physiological and psychological mechanisms. Using, for instance, Transcranial Magnetic Stimulation (TMS), it is possible to produce very focal electric field patterns (Deng et al., 2013) and therefore explicitly stimulate a circumscribed target region. This is particularly helpful in research fields where certain brain areas (as parts of respective brain networks) have repeatedly been found to show aberrant functioning in patients.

There are various findings on prefrontal hypoactivation in patients with depression (DP) within the left Dorsolateral Prefrontal Cortex (DLPFC) during “affective and cognitive tasks requiring emotional or stress regulation, cognitive control, and/or shifting attention to external task demands” (page 246, Pizzagalli & Roberts, 2022). This is currently thought to be due to reduced recruitment of the DLPFC in general, as well as cortical inefficiency in DP. Cortical inefficiency in this context refers to the idea that the DLPFC, even when recruited, fails to engage effectively when needed. This could mean that greater neural activity is required to achieve the same level of cognitive or emotional regulation as in healthy controls. Consequently, whereas healthy controls and DP show similar DLPFC activation at some point, an increased need for resources (e.g., in case of higher stress) leads to aberrant functioning both on a neural as well as behavioral level in DP. What is particularly interesting, is that studies including experimental stress inductions, such as the Trier Social Stress Test (TSST; Kirschbaum et al., 1993), which is a very potent and ecologically valid stressor (Allen et al., 2017; Henze et al., 2017, 2023; Kudielka et al., 2007), have found that prefrontal hypoactivation under stress was not only observed in DP (Rosenbaum et al., 2021) but also in healthy controls with an increased tendency to ruminate (Rosenbaum, Hilsendegen et al., 2018a, Rosenbaum, Thomas et al., 2018b). This gives rise to the question to what extent ruminative thinking, which has been found to be induced by using stress inductions, such as the TSST, might be associated with a somewhat inefficient recruitment of brain regions involved in cognitive control (e.g., the DLPFC as part of the Central Executive Network) and therefore result in insufficient inhibition of negative, self-focused, and nongoal-oriented thoughts. “Rumination is repetitive, prolonged, and recurrent negative thinking about one's self, feelings, personal concerns and upsetting experiences” (page 1, Watkins & Roberts, 2020). Ruminative thinking is also apparent in healthy controls but has been recognized as a significant risk factor for the onset of mood disorders, including depression and anxiety, as well as other mental health conditions. As such, it is regarded as a transdiagnostic cognitive vulnerability (Nolen-Hoeksema et al., 2008). Ruminative thinking is associated with increased and prolonged negative affect, stress and has been shown to interfere with effective problem-solving, because it has little to no goal orientation (for comprehensive reviews, see Nolen-Hoeksema et al., 2008; Watkins & Roberts, 2020). However, rumination affects not only the psychological but also the physiological stress response, as a meta-analysis has shown its association with elevated blood pressure, increased heart rate, reduced heart rate variability, and higher cortisol levels (Ottaviani et al., 2016).

First investigations regarding the neural underpinnings of rumination have found primarily greater activation and connectivity within regions of the Default Mode Network, which is associated with increased self-referential thinking (e.g., amygdala, medial prefrontal cortex, posterior cingulate cortex and precuneus) (Berman et al., 2011; Cooney et al., 2010; Hamilton et al., 2011, 2015; Jacob et al., 2020; Jones et al., 2017; Mandell et al., 2014; Murphy et al., 2016; Nejad et al., 2013; Philippi et al., 2018; Ray et al., 2005; Siegle et al., 2002). Moreover, prefrontal regions, such as the DLPFC and VLPFC, have consistently been shown to exhibit altered functioning in response to ruminative thinking (Cooney et al., 2010; Kühn et al., 2014; Ray et al., 2005; Rosenbaum, Thomas et al., 2018b, Rosenbaum et al., 2021).

In this context, TMS is capable of inducing excitability changes in the cortex and investigating the causal involvement of the corresponding brain networks. There are various forms of TMS, such as single-pulse, paired pulse, or repetitive TMS (rTMS). The latter is found to produce longer-lasting effects, persisting past the period of stimulation (Oberman, 2014). Recently, rTMS has been intensely studied, for instance, in the context of the treatment of depression by modulating activation of the left DLPFC (Gaynes et al., 2014; Sathappan et al., 2019; Schutter, 2009). In experimental settings, rTMS has also been found to be capable of examining effects on rumination and also the psychophysiological stress response (Baeken et al., 2014; De Smet et al., 2021; Pulopulos et al., 2020; Remue et al., 2016). A relatively newer variant of rTMS, Theta Burst Stimulation (TBS), in which high-frequency TMS is applied in the form of bursts simulating the theta-rhythms of the hippocampus (Huang et al., 2005), offers particularly interesting possibilities. Specifically, depending on the interpulse interval and intertrain interval, TBS is capable of eliciting excitatory effects that are assumed to resemble long-term potentiation (intermittent Theta Burst Stimulation; iTBS) or inhibitory effects resembling long-term depression of neural excitability (continuous Theta Burst Stimulation; cTBS) (Huang et al., 2011). So far, only three studies employed TBS in the context of stress. Pulopulos et al. (2019) were the first to investigate iTBS on the left DLPFC and found no general impact on mood or cortisol responses. This was replicated by De Witte et al. (2020), who also found no significant effect of iTBS on mood or cortisol. However, they observed that trait rumination was linked to increased rumination only in the sham condition, suggesting that iTBS may disrupt the relationship between brooding and stress-reactive rumination, potentially regulating the stress response. Era et al. (2021) applied cTBS to the left DLPFC and found that it induced increased heart rate and cortisol responses, indicating a detrimental, stress-reactive effect. Overall, these studies point to the complexity of the relationship between TBS, stress, and rumination, suggesting a need for further research, particularly with larger samples and neural data. Including both state and trait rumination as variables could be promising for understanding these effects.

In combination with the finding that the left DLPFC is considered the most appropriate target region for manipulating stress reactivity using NIBS (Moses et al., 2023), the aim of the current study was to evaluate the effects of cTBS and iTBS versus sTBS on the neural correlates of stress-reactive rumination and the psychophysiological stress response. The neural correlates were investigated using functional near-infrared spectroscopy (fNIRS), which has been found capable of assessing TBS-induced changes in cortical oxygenation in the DLPFC in previous studies (Curtin et al., 2019).

We hypothesized that inhibiting the left DLPFC with cTBS would exacerbate prefrontal dysfunction under stress in high ruminators, whereas excitatory stimulation (iTBS) would “normalize” cortical oxygenation in the left DLPFC, leading to higher oxygenation. For low ruminators, we expected inhibitory stimulation to decrease cortical oxygenation, while excitatory stimulation would increase it, although the effect would likely be smaller, as low ruminators are thought to already have higher prefrontal activity under stress. For the fNIRS data, we anticipated higher cortical oxygenation in the left DLPFC following iTBS compared with sTBS and lower oxygenation after cTBS compared with sTBS during the stress induction (i.e., the arithmetic task of the TSST). We predicted that iTBS would reduce subjective stress, negative affect, and stress-reactive rumination, while cTBS would increase these measures. However, the effects of iTBS in low ruminators were expected to be modest owing to their already high prefrontal functioning and low rumination. We expected the TBS effects to last at least through the TSST (approximately 20 min post TBS) and the second resting-state measurement (approximately 30 min post TBS).

## Methods

### Participants

**Fig. 1:**
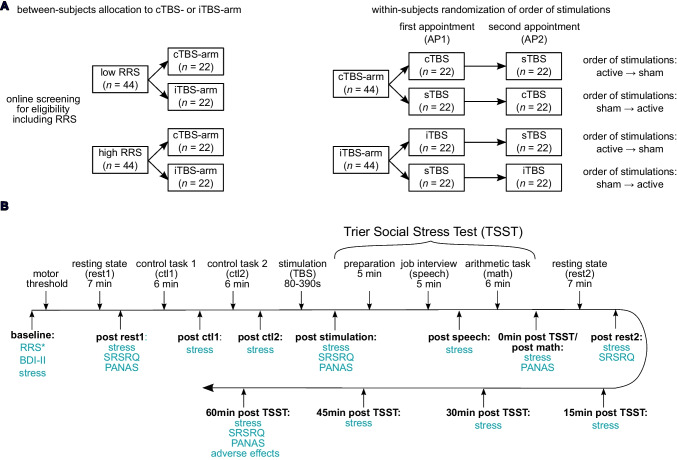
The allocation of experimental groups (**A**) and overview of the experimental procedure (**B**). Above the line, the respective tasks and experimental phases are shown. Below the line, the time points of questionnaire assessments are displayed in bold, with the names of the corresponding questionnaires highlighted in blue. RRS = Ruminative Response Scale; TBS = Theta Burst Stimulation; BDI-II = Beck's Depression Inventory II; PANAS = Positive and Negative Affect Schedule; SRSRQ = Stress-Reactive State Rumination Questionnaire; stress = subjective stress assessed via a Visual Analogue Scale (0–100%); *Only assessed at the first appointment (AP1)

Interested volunteers were made aware of the study by means of circular emails via the university mailing list. Initially, an eligibility screening using an online questionnaire was completed, in which demographic and clinical variables as well as the Ruminative Response Scale (RRS; Treynor & Gonzalez, 2003) were assessed (for a list of inclusion and exclusion criteria see supplementary material S1). According to an a priori power analysis (see supplementary material S2), we aimed to recruit a stratified sample of 44 low and 44 high trait ruminators (low trait ruminators: mean RRS ≤ 1.82 (PR 25); high trait ruminators: mean RRS ≥ 2.36 (PR 64). Please note that these corresponding cutoffs are based on the combined data of 983 participants from prior studies (Rosenbaum, Hilsendegen et al., 2018a, Rosenbaum, Thomas et al., 2018b, Rosenbaum et al., 2021). Following the a priori power analysis, we recruited participants until this target was reached, which resulted in the final sample size matching the planned number. All eligible volunteers received an invitation to participate in the study. After inclusion in the study, a total of 22 participants declined to participate (*n* = 15 owing to loss of interest in participation prior to the first appointment, *n* = 2 owing to discomfort with the TBS, *n* = 2 owing to circulatory problems during the measurement,* n* = 2 without giving a reason, and *n* = 1 owing to discomfort of the stress induction). A total of seven participants were excluded because their mean RRS substantially changed between the online screening and a second assessment (second online screening 1 week prior to the lab appointment or at the first lab appointment) either by changing the category or falling between the cutoffs and being closer to the corresponding other category^1^ (please find the CONSORT-diagram in supplementary material S3). In total, the final sample comprised 88 right-handed healthy volunteers aged 18 to 50 years (44 low and 44 high trait ruminators). After inclusion, participants were randomly assigned to one of two stimulation conditions: iTBS (*n* = 44; 22 low ruminators, 22 high ruminators) or cTBS (*n* = 44; 22 low ruminators, 22 high ruminators). Each participant completed two laboratory sessions (AP1 and AP2), during which they received either an active (iTBS or cTBS) or a sham (sTBS) stimulation. The sham condition was matched in duration to its corresponding active stimulation (i.e., participants receiving active cTBS underwent sTBS of the same duration as cTBS, and those receiving active iTBS underwent sTBS of the same duration as iTBS). The order of stimulation (active vs. sham) was randomized and counterbalanced across participants and stimulation groups (for an illustration of the allocation of experimental groups, see Fig. 1A).

### Procedure

After inclusion in the study, participants underwent two appointments at the laboratory. At their first appointment, participants gave written informed consent. Afterwards, at each appointment, the participants’ resting motor threshold was determined using the visual observation of muscle twitch with the relative frequency method (Rothwell et al., 1999). While participants were prepared for the electrocardiogram (ECG)- and fNIRS-measurement, they completed several questionnaires assessing demographic data, symptoms of depression (Beck’s Depression Inventory II: BDI-II; Beck et al., 1996) and trait rumination (Ruminative Response Scale: RRS; Treynor & Gonzalez, 2003) and a Visual Analogue Scale assessing subjective stress levels on a scale ranging from 0% to 100%. We do not anticipate any significant influence of the preparation process on the questionnaire responses, as participants were only briefly interrupted during the completion of the questionnaires and were not under direct observation. Then, a 7 min resting-state measurement followed (rest1), where participants were instructed to let their mind wander but keep their eyes open. Following this, participants gave another stress rating and completed a questionnaire assessing their current affect (Positive and Negative Affect Schedule: PANAS; Watson et al., 1988; please note that we used an adapted version with two additional items, namely “happy” and “sad”) and an assessment of state rumination using the Stress-Reactive State Rumination Questionnaire (SRSRQ; Int-Veen, Laicher et al., n.d b for a full list of items, see supplementary material S4). Following this, participants completed two control tasks as in Rosenbaum, Hilsendegen et al. (2018a), Rosenbaum, Thomas et al. (2018b), and Rosenbaum et al. (2021) including reading numbers aloud (control task 1; ctl1) and performing mental arithmetic without time or social pressure (control task 2; ctl2). Both tasks consisted of 6 trials of 40 s duration and 20 s rest, and subjective stress ratings were assessed following each task. Then participants were guided to another room where the neurostimulation followed (see the section on Theta Burst Stimulation). After the TBS and another stress rating, as well as an assessment of the PANAS and SRSRQ, participants underwent the stress induction (see section Trier Social Stress Test). Following the TSST (0 min post TSST), participants again rated their current stress and completed another PANAS prior to another 7 min resting-state measurement (rest2). Finally, subjective stress and another SRSRQ were assessed. For the last part of the study, participants were again guided to the room where the TBS took place where they were seated until 60 min post TSST and instructed to let their mind wander, while another stress rating was assessed every 15 min. At 60 min post TSST, participants additionally completed a PANAS, SRSRQ and a questionnaire about potential side effects of the TBS. Lastly, as a manipulation check, we asked participants about their beliefs regarding the stimulation condition they were in (sham vs. active) and how confident they were in answering the question. After completion of both appointments, participants were debriefed and received 100 € as monetary compensation or 6 hours of course credit (for an overview of the study procedure, see Fig. 1B). The two appointments (AP1 and AP2) at the laboratory were scheduled approximately 5 weeks apart (*M* = 41.48 days, *SD* = 8.45 days).

This study was approved by the ethics committee at the University Hospital and University of Tübingen (673/2019BO1).

### Theta Burst Stimulation

The motor threshold determination and stimulation were performed by a study nurse who was no further involved in the study. We used a MagVenture MagPro X100 Stimulator (MagVenture, Farum, Denmark) as well as a figure-eight shaped coil without cooling for motor threshold determination (MagVenture C-B60 coil) and another figure-eight shaped coil with active cooling (MagVenture Cool-B65 Active/Placebo coil), specifically designed for double-blind stimulation by automatically changing between active or sham TBS via coil flipping according to the device instructions. A total of 88 pairs of randomization codes were created, with each participant assigned one pair. One code in the pair triggered active stimulation, and the other triggered sham stimulation in the TBS device. The order of stimulation (active → sham or sham → active) was balanced across participants. The first code in the pair was used for appointment 1 (AP1), and the second code for appointment 2 (AP2). The randomization was conducted using Excel prior to the study. Randomization pairs were assigned in the order of participant recruitment. To ensure balanced randomization across the RRS-groups (low and high ruminators), numbers 1 to 4 representing the four conditions were listed 11 times each and then randomly shuffled. The four conditions were: “cTBS-arm: active → sham”; “cTBS-arm: sham → active”; “iTBS-arm: active → sham”; and “iTBS-arm: sham → active”.

TBS over the left DLPFC was applied at 80% of the resting motor threshold (Huang et al., 2005). The coil was positioned according to the 10–20 electrode position F3 (Jasper, 1958), which corresponded to one channel of the left frontal fNIRS probeset (for an illustration of the TBS-induced electric field, see supplementary material S5). Please note that the coil was placed directly on the scalp, as the fNIRS device did not permit simultaneous acquisition of fNIRS data and TBS. To mask the active stimulation and ensure better blinding in the case of sTBS, two pre-gelled surface electrodes (28 x 20 mm) were placed 1 cm around the stimulation site applying a low current for a superficial sensation during the TBS. The sham side of the coil is shielded, preventing the transmission of magnetic pulses to the brain. As a result, while participants still experience the typical auditory effects of stimulation, they do not perceive any tactile sensation. The additional surface electrodes were introduced to ensure a more effective blinding procedure by providing a superficial sensory experience during sTBS. However, the applied current is minimal and did not penetrate the brain, nor did it stimulate peripheral nerves (Smith & Peterchev, 2018). We opted for this method to enhance blinding and minimize potential differences in subjective experience between active and sham stimulation, thereby reducing the risk of unblinding effects that could influence neural and behavioral responses. Participants were randomly assigned to receive cTBS (80 s train of uninterrupted TBS including 400 bursts of 3 pulses at a frequency of 50 Hz and burst frequency of 5 Hz) or iTBS (40 cycles of 2 s theta burst trains (10 bursts of 3 pulses each) followed by 8 s of rest (i.e., total of 390 s). Consequently, the stimulation parameters included a total of 1,200 pulses for both paradigms.

### Trier Social Stress Test

To reliably induce psychosocial stress following the TBS, we used an adapted version of the Trier Social Stress test (TSST; Kirschbaum et al., 1993). After the participants had been guided back to the measurement room and were prepared for the ECG- and fNIRS-measurement, they were told that two other people were going to join. Two experimenters wearing white physician coats entered and took a seat in front of the participant's table. They instructed the participant to imagine having applied for a job at the university hospital, with part of the recruitment procedure involving a speech about their personal strengths and qualification for the corresponding position. Participants had 5 min to prepare for this speech by taking notes, which were then collected. Standing up in front of the two experimenters, who remained unresponsive to any social interaction signs, participants were urged to talk for 5 min without this time limit being known beforehand. During this, participants were video recorded in order to maximize social threat. After the 5 min speech, participants completed a subjective stress rating receiving instructions about the next task. For the mental arithmetic task, participants were instructed to calculate aloud as quickly and accurately as possible from various starting points (e.g., 1013). Additionally, participants were instructed to maintain eye contact with one experimenter while the other experimenter asked them to start over if they made an error. Similar to the control tasks, participants completed 6 trials of 40 s each, with 20 s pauses in order for the hemodynamic response to recover. After the final trial, the experimenters stopped the video recording and left the room without any comment.

### Electrocardiogram

Throughout the experimental session (rest1 until rest2), heart rate was assessed by using three Ag/AgCl ring electrodes with a diameter of 8 mm, which were attached to the participants'skin above the right collar bone, below the left costal arch and below the neck (reference). The signal was recorded with a BrainAmp ExG amplifier and Brain Vision recorder software (Brain Products, Munich, Germany) at a sampling rate of 1,000 Hz. Data were preprocessed and analyzed by using Brain Vision Analyzer 2.1 and MATLAB 2024. Preprocessing included band-pass filtering (1–30 Hz; slope: 48 db/Oct, time constant 0.1591549 s) and applying a notch filter at 50 Hz to eliminate power line artifacts. Lastly, we calculated the mean interval between subsequent R-peaks for each recorded condition in beats per minute (BPM).

### Functional Near-Infrared Spectroscopy

Throughout the experimental session (rest1 until rest2), cortical oxygenation was measured using an ETG-4000 Optical Topography System with a sampling rate of 10 Hz (46-channel continuous wave multichannel fNIRS; Hitachi Medical Co., Japan). Two frontal and one parietal probeset with a fixed 3 cm inter-optode distance (28 light emitters, i.e., semiconductor lasers and 15 light detectors, i.e., avalanche photodiodes at two wavelengths (695 ± 20 and 830 ± 20 nm) with 2.0 ± 0.4 mW for each wavelength at each optode) were placed according to the 10–20 reference points Fpz and Cz. Relative changes in oxygenated (O_2_Hb) and deoxygenated (HHb) hemoglobin were computed using custom MATLAB 2024 scripts by means of the modified Beer-Lambert Law (Sassaroli & Fantini, 2004). Preprocessing included interpolation of single noisy channels, correction of motion artifacts using Temporal Derivative Distribution Repair (Fishburn et al., 2019), Correlation-based signal improvement (Cui et al., 2010) and bandpass filtering to remove low-frequency baseline drifts (< 0.01 Hz) and high-frequency noise (> 0.1 Hz). To remove artifacts due to data correction, additional channel interpolation was performed, followed by a global signal reduction with a spatial gaussian kernel filter (σ = 40) and z-standardization of the signal. Note that in the following, O_2_Hb data refers to the correlation-based improved O_2_Hb signal. For data analysis, we calculated event-related averages including a 5 s baseline correction. For a visualization of the probeset placement and respective Regions of Interest (ROI), we refer to supplementary material S6 and S7. Lastly, data was exported as an average for each ROI as well as each channel separately: left and right Ventrolateral Prefrontal Cortex (VLPFC), left and right Dorsolateral Prefrontal Cortex (DLPFC), and Somatosensory Association Cortex (SAC). Scalp-brain correspondence was estimated based on Okamoto et al. (2004), Okamoto & Dan (2005) and Singh et al. (2005). Please note that channel 12 of the left frontal probeset, which corresponds to electrode position F3 of the 10–20 system, was the stimulated site.

### Data analysis

Data analysis was conducted by using SPSS (Version 28, IBM Corp., 2021).

To analyze the effects of TBS, we applied three complementary analytical approaches for all dependent variables (DV): subjective stress, state rumination, positive and negative affect, math performance, heart rate, and cortical oxygenation (to reduce the article's length, results on positive affect, math performance, and heart rate are included in supplementary material S8).

First, following standard procedures for within-subject designs, we conducted contrast analyses comparing active stimulation (iTBS/ cTBS) to sham stimulation. For this, we calculated contrasts for each DV by subtracting sham stimulation values from active stimulation values at each time point. These contrasts were then entered into repeated measures ANOVAs (rmANOVAs), including a four-way interaction of time, stimulation condition (iTBS vs. cTBS), group (low RRS vs. high RRS), and order of stimulation conditions (active → sham vs. sham → active). Please note that “time” refers to the number of repeated assessments of the corresponding DVs (12 times for subjective stress, 4 times for state rumination, 4 times for positive and negative affect, 3 times for math performance, 7 times for heart rate, and 3 times for cortical oxygenation).

We identified strong habituation effects in nearly all DVs, which is in line with previous findings of habituation effects on the HPA-axis response with repeated exposure to the TSST (Allen et al., 2017; Kudielka et al., 2007; Schommer et al., 2003). Most probably owing to the novelty of the situation, stress responses were stronger during the initial exposure, which might cause the small TBS effects to be masked. After an initial a priori planned analysis of the rmANOVAs without the factor “order of stimulation conditions” yielding predominantly null results, we decided to include the factor in the rmANOVA, because incorporating the order of stimulation conditions as a factor enables a more precise understanding of how habituation interacts with the effects of the stimulation on stress reactivity. If the highest interaction involved the order of stimulation conditions, separate rmANOVAs were performed based on the order, excluding lower-order effects to streamline the results.

In addition to the contrast analysis, we reported the raw data to provide a clearer overview of the observed effects. More specifically, we fitted rmANOVAs including a three-way interaction of time, stimulation condition (iTBS vs. cTBS vs. sTBS), group (low RRS vs. high RRS) for each appointment, separately.

Finally, we conducted planned contrasts to examine specific time points at which TBS effects were expected to be most pronounced. This approach provided a more targeted hypothesis test while increasing statistical power.

For each analysis, multivariate outliers were identified using Mahalanobis distances separately for each DV. Please note that there were no significant baseline differences between the stimulation conditions.

For fNIRS data specifically, we further performed a manipulation check in the form of paired *t*-tests to compare the cortical oxygenation in each channel during the arithmetic task of the TSST, contrasting the levels after active stimulation with those following sham stimulation (active vs. sham) within subjects across all participants. Then, a repeated measures MANOVA (rmMANOVA) was conducted based on five ROIs (left VLPFC, left DLPFC, right VLPFC, right DLPFC, and SAC), including the aforementioned four-way interaction.

Lastly, we calculated Reliable Change Indices (RCI) for state rumination ratings to determine how many participants exhibited reliable changes in their ratings between post rest1 and post rest2. We calculated RCI to assess whether the TSST has led to significant changes at the individual level, complementing group-level statistical analyses (for further reading, see Blampied, 2022).

Significant effects were followed by post-hoc tests corrected for multiple comparisons using the Benjamini-Hochberg procedure. Nonsignificant post-hoc tests and those related to lower-order effects involved in significant higher-order interactions are not reported. Polynomial contrasts (linear and quadratic) were provided for interpretative purposes. Please note that we also investigated quadratic contrasts because this approach captures potential nonlinear changes, such as an increase or decrease during stress induction followed by a return toward baseline in the post-stress phase.

As an exploratory analysis, we also examined the influence of expectancy effects on the previously described analyses. For this purpose, we included the item “Do you believe that the stimulation made you perform better or worse on the task? (better vs. worse vs. no effect)” as a covariate.

All significant effects, including lower-order ones, are reported, and marginally significant effects are noted if *p* < .1. Violations of sphericity (Mauchly test *p* < .05) were corrected using Greenhouse-Geisser estimates if *ϵ* < 0.75, and Huynh-Feldt estimates if *ϵ* > 0.75.

Data visualization was conducted using MATLAB 2024, RStudio Version 2022.02.3+492 (Studio Team, 2022), R Version 4.3.1 (R Core Team, 2023), and the ggplot2 package (Wickham, 2016).

## Results

### Sample characteristics

Overall, the average age of the sample was 24.22 years (*SD* = 4.85 years), and 79.55% of the participants were female. On average, participants had a mean RRS score of 1.99 (*SD* = 0.64) and a BDI-II total score of 7.22 (*SD* = 7.19), indicating “no depression” according to the BDI-II cutoff scores (Hautzinger et al., 2009). There were no significant differences in demographic variables between the cTBS- and iTBS study arm (see Table 1).

**Table 1:** Demographic variables of the sample by study arm

	cTBS-arm	iTBS-arm	Test statistic	Total sample
Age	24.05 (2.91)	24.50 (6.30)	*F*(1, 86) = 0.255, *p* = .615, *η*_*p*_^*2*^ = .003	24.22 (4.85)
Percent female	77.27%	81.82%	${\upchi }^{2}$(1) = 0.070, *p* = .792	79.55%
BDI-II	6.91 (7.04)	7.53 (7.91)	*F*(1, 86) = 0.153, *p* = .697, *η*_*p*_^*2*^ = .002	7.22 (7.19)
RRS	2.04 (0.68)	1.93 (0.60)	*F*(1, 86) = 0.723, *p* = .398, *η*_*p*_^*2*^ = .008	1.99 (0.64)

BDI-II = Beck Depression Inventory II; RRS = Rumination Response Scale. Test statistic = comparison of the cTBS- and iTBS-arm

### Manipulation check and blinding

A binomial test showed that participants could not distinguish between sham and active stimulation, as correct guesses in all stimulation conditions were not significantly different from chance (all *p's* > .101; see Table 2). Additionally, there were no significant differences in stimulation intensity among the cTBS (*M* = 42.34, *SD* = 6.10), iTBS (*M* = 41.11, *SD* = 7.05), and sTBS conditions (*M* = 41.83, *SD* = 6.24), *F*(2, 173) = 0.408, *p* = .666, *η*_*p*_^*2*^ = .005.

**Table 2:** Summary of exact binomial tests assessing the blinding of participants (${H}_{0}$: *p* = .5)

	Percent	95% CI	*p*
Correct identification of sTBS as sham (AP1)	56.10%	[39.75; 71.53]	.533
Correct identification of iTBS as active (AP1)	50.00%	[27.20; 72.80]	.999
Correct identification of cTBS as active (AP1)	38.10%	[18.11; 61.57]	.383
Correct identification of sTBS as sham (AP2)	62.79%	[46.73; 77.02]	.126
Correct identification of iTBS as active (AP2)	66.67%	[43.03; 85.41]	.189
Correct identification of cTBS as active (AP2)	63.64%	[40.66; 82.80]	.286
Correct identification of sTBS as sham (overall)	59.52%	[48.25; 70.10]	.101
Correct identification of iTBS as active (overall)	58.54%	[42.11; 73.68]	.349
Correct identification of cTBS as active (overall)	51.16%	[35.47; 66.69]	.999

*AP1* first appointment; *AP2* second appointment; *sTBS* sham Theta Burst Stimulation; *cTBS* continuous Theta Burst Stimulation; *iTBS* intermittent Theta Burst Stimulation

We examined whether participants believed that stimulation affected their performance (either improving or worsening it) using a logistic regression model. The results showed no significant effect of the stimulation condition, ${\upchi }^{2}$ (2) = 2.492, *p* = .288.

Next, we used a linear mixed model to analyze participants' confidence in their responses to a question about the impact of stimulation on their performance. More specifically, we fitted a model with the response to the respective item as DV, stimulation condition as a predictor and separate intercepts per subject. Again, we found no significant main effect of the condition, ${\upchi }^{2}$ (2) = 1.946, *p* = .378. Descriptively, in all three TBS groups, approximately half of the participants felt that stimulation had no impact on their performance, whereas the other half believed it did (see Table 3).

**Table 3:** Absolute and relative frequencies of participants' ratings on the impact of stimulation on performance by stimulation condition

			Condition			total
			sTBS	cTBS	iTBS	
Impact on performance	No change	count	45	17	25	87
		% within impact on performance	51.7%	19.5%	28.7%	100%
		% within condition	52.9%	41.5%	58.1%	51.5%
		% of all data points	26.6%	10.1%	14.8%	51.5%
	Better	count	23	15	9	47
		% within impact on performance	48.9%	31.9%	19.1%	100%
		% within condition	27.1%	36.6%	20.9%	27.8%
		% of all data points	13.6%	8.9%	5.3%	27.8%
	Worse	count	17	9	9	35
		% within impact on performance	48.6%	25.7%	25.7%	100%
		% within condition	20.0%	22.0%	20.9%	20.7%
		% of all data points	10.1%	5.3%	5.3%	20.7%

*sTBS* sham Theta Burst Stimulation; *cTBS* continuous Theta Burst Stimulation; *iTBS* intermittent Theta Burst Stimulation. Please note that 7 data points were missing as the item was not answered or not answered unambiguously

Additionally, when analyzing the logistic regression and linear mixed models based on the RRS group, there were no significant effects of the group, ${\upchi }^{2}$ (1) = 0.980, *p* = .322 and ${\upchi }^{2}$ (1) = 0.629, *p* = .428, respectively. For both RRS groups, most participants thought that stimulation had no effect (low RRS group: 47.6% no change, 30.5% better, 22.0% worse; high RRS group: 55.2% no change, 25.3% better, 19.5% worse).

### Psychological measures

#### Subjective stress contrasts

We identified multivariate outliers using Mahalanobis distances (*p* < .001) and excluded the data from 5 participants for the subsequent analysis.

When we fitted the rmANOVA of the subjective stress contrasts, we observed a significant four-way interaction between time, group, stimulation condition, and order of stimulation conditions, *F*(5.005, 375.371) = 3.338, *p* < .01, ${\eta }_{p}^{2}$ = .043, as well as significant lower-order interactions: a time and order of stimulation conditions interaction, *F*(5.005, 375.371) = 23.818, *p* < .001, ${\eta }_{p}^{2}$ = .241; a three-way interaction of group, stimulation condition, and order of stimulation conditions, *F*(1, 75) = 6.166, *p* < .05, ${\eta }_{p}^{2}$ = .076; an interaction between group and stimulation condition, *F*(1, 75) = 4.418, *p* < .05, ${\eta }_{p}^{2}$ = .056; and a main effect of the order of stimulation conditions, *F*(1, 75) = 37.940, *p* < .001, ${\eta }_{p}^{2}$ = .336.

To gain a deeper understanding of the four-way interaction, we performed separate rmANOVAs considering the order of the stimulation conditions. As a result, only for participants who received sham first, we observed a significant three-way interaction of time, group and stimulation condition, *F*(4.806, 182.645) = 3.805, *p* < .01, ${\eta }_{p}^{2}$ = .091. Additionally, there was a significant intercept regardless of the order of stimulation conditions (i.e., a significant main effect of active stimulation vs. sham) (sham → active: *F*(1, 38) = 21.698, *p* < .001, ${\eta }_{p}^{2}$ = .363; active → sham: *F*(1, 37) = 17.318, *p* < .001, ${\eta }_{p}^{2}$ = .319).

**Fig. 2:**
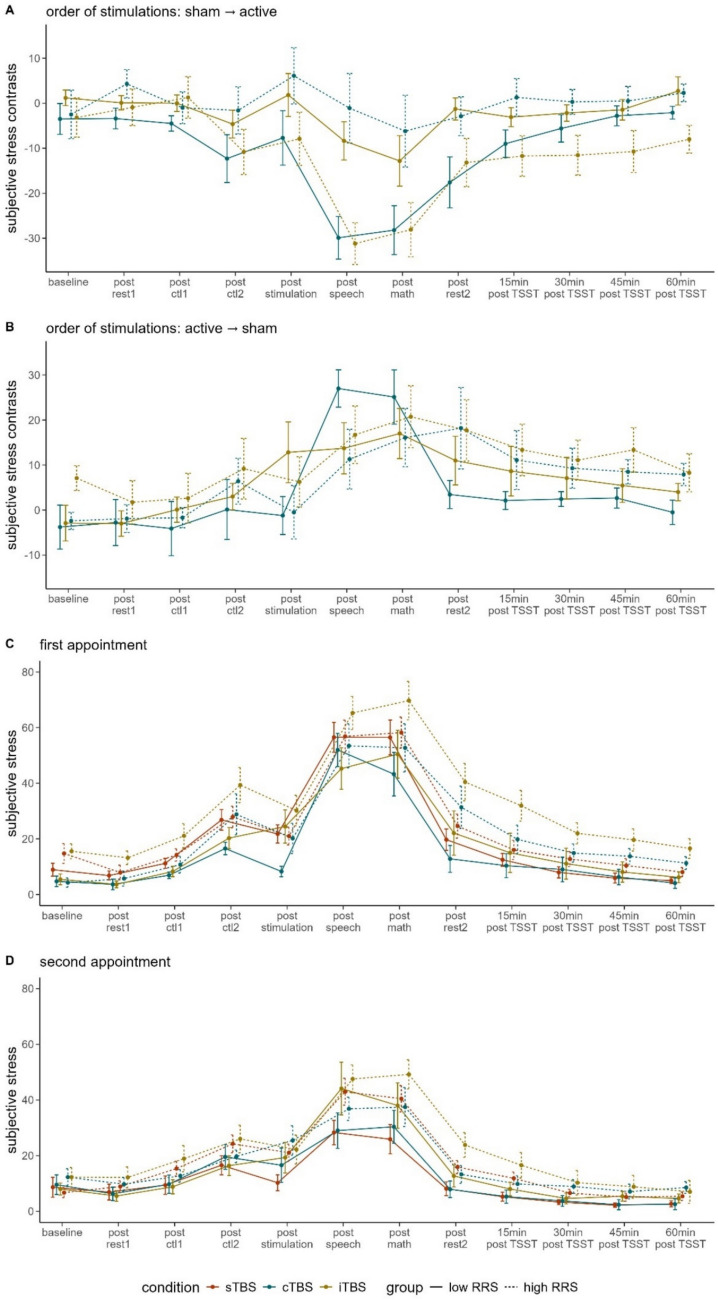
Line plot of the contrasts of subjective stress ratings dependent on order of conditions (**A** = sham stimulation at the first appointment, active stimulation at the second appointment; **B** = active stimulation at the first appointment, sham stimulation at the second appointment), and line plot of raw data of subjective stress ratings dependent on the appointment (**C** = first appointment; D = second appointment). rest = resting-state measurement; ctl1 = control task 1; ctl2 = control task 2; TSST = Trier Social Stress Test; sTBS = sham Theta Burst Stimulation; cTBS = continuous Theta Burst Stimulation; iTBS = intermittent Theta Burst Stimulation; 15 min = 15 min after the TSST; RRS = Ruminative Response Scale. Please note that the effect of the order of stimulation conditions is visually depicted within the plots of the different appointments (first appointment: sTBS = participants with order of stimulation conditions sham → active; iTBS and cTBS = participants with order of stimulation conditions active → sham; second appointment: vice versa). Error bars indicate 1 standard error of the mean

Polynomial contrasts of the three-way interaction of time, group and stimulation condition revealed a significant quadratic contrast, *F*(1, 38) = 12.630, *p* < .01, ${\eta }_{p}^{2}$ = .249, and post-hoc tests indicated significant differences between stimulation conditions in high ruminators following the job interview of the TSST. Specifically, high ruminators exhibited more negative contrasts following cTBS (*M* = −1.10, *SD* = 24.42) compared to high ruminators following iTBS (*M *= −31.18, *SD* = 15.43), *t*(19) = 3.410, *p* < .01, *d* = 1.490. In both cases, we observed negative contrasts, indicating higher subjective stress following the job interview of the TSST in case of the sham compared to the active appointment (i.e., the first appointment, AP1) but more negative contrasts following iTBS (i.e., more pronounced differences between active and sham stimulation). The raw data shows that high ruminators who received sTBS of the duration of iTBS at AP1 generally reported higher levels of subjective stress (*M* = 80.60, *SD* = 12.14) compared with high ruminators receiving sTBS of the duration of cTBS at AP1 (*M* = 38.73, *SD* = 19.54), *t*(18.171) = −5.529, *p* < .001, *d* = −2.260. At AP2, both groups exhibited significantly lower stress (*M*_*iTBS*_ = 47.55, *SD*_*iTBS*_ = 16.68; *M*_*cTBS*_ = 35.50, *SD*_*cTBS*_ = 13.83), whereas the group receiving iTBS exhibited stronger and the cTBS-group non-significant reductions (iTBS-arm: *t*(19) = 5.144, *p* < .001, *d* = 2.248; cTBS-arm: *t*(19) = 0.433, *p* = .335, *d* = 0.189) (Fig. 2).

#### Subjective stress raw data

Based on Mahalanobis distances (*p* < .001), we excluded the data from eight participants: three from the first appointment (AP1) and five from the second appointment (AP2).

When we fitted the rmANOVA separately for each appointment on the raw data, we observed a significant main effect of time (AP1: *F*(3.458, 273.160) = 137.998,* p* < .001, ${\eta }_{p}^{2}$ = .636; AP2: *F*(2.985, 229.849) = 93.942, *p* < .001, ${\eta }_{p}^{2}$ = .550) with quadratic polynomial contrasts each (AP1: *F*(1, 79) = 252.274, *p* < .001, ${\eta }_{p}^{2}$ = .762; AP2: *F*(1, 77) = 149.649, *p* < .001, ${\eta }_{p}^{2}$ = .660), indicating increases in subjective stress due to the TSST and respective decrease post-stress (see Fig. 2C-D).

We further observed a significant main effect of group at both appointments, indicating that high ruminators reported significantly higher subjective stress compared to low ruminators (AP1: *F*(1, 79) = 10.791,* p* < .01, ${\eta }_{p}^{2}$ = .120; AP2: *F*(1, 77) = 8.315, *p* <.01, ${\eta }_{p}^{2}$ = .097).

Lastly, polynomial contrasts indicated a significant linear contrast for the interaction of time and stimulation condition in case of the first appointment, *F*(2, 79) = 4.079, *p* < .05, ${\eta }_{p}^{2}$ = .094, and a marginally significant quadratic contrast of the interaction of time and group, indicating higher stress-induced increases in high ruminators, *F*(1, 77) = 3.156, *p* = .080, ${\eta }_{p}^{2}$ = .039.

#### Subjective stress planned contrasts

When we investigated the differences in subjective stress separately for each appointment and RRS group, we observed significantly higher subjective stress in high ruminators at their first appointment following iTBS compared with high ruminators at their first appointment following sTBS after the second resting-state measurement, *t*(29) = −2.065, *p* < .05, *d* = −0.775 (*M*_*sTBS*_ = 24.60, *SD*_*sTBS*_ = 19.35, *M*_*iTBS*_ = 40.46, *SD*_*iTBS*_ = 22.41), 15 min, *t*(29) = −2.575, *p* < .01,* d* = −0.967 (*M*_*sTBS*_ = 16.05, *SD*_*sTBS*_ = 15.28, *M*_*iTBS*_ = 31.91, *SD*_*iTBS*_ = 18.37), 30 min,* t*(29) = −1.988, *p* < .05, *d* = −0.746 (*M*_*sTBS*_ = 12.75, *SD*_*sTBS*_ = 12.21, *M*_*iTBS*_ = 22.00, *SD*_*iTBS*_ = 12.73), 45 min,* t*(29) = −2.061, *p* < .05, *d* = −0.773 (*M*_*sTBS*_ = 10.40, *SD*_*sTBS*_ = 11.17, *M*_*iTBS*_ = 19.64, *SD*_*iTBS*_ = 13.29), and 60 min after TSST, *t*(29) = −2.437,* p* < .05, *d* = −0.915 (*M*_*sTBS*_ = 8.05, *SD*_*sTBS*_ = 7.51, *M*_*iTBS*_ = 16.55, *SD*_*iTBS*_ = 11.96).

We further observed significantly higher subjective stress in high ruminators at AP2 following iTBS compared with high ruminators at AP2 following sTBS but only after the second resting-state measurement, *t*(29) = −1.743, *p* < .05, *d* = −0.654 (*M*_*sTBS*_ = 15.90, *SD*_*sTBS*_ = 10.45, *M*_*iTBS*_ = 23.82, *SD*_*iTBS*_ = 14.74) as well as marginally significantly higher subjective stress in low ruminators at AP2 following iTBS compared with high ruminators at AP2 following sTBS but only 45 min after the TSST, *t*(12.385) = −1.685, *p* = .058, *d* = −0.768 (*M*_*sTBS*_ = 2.20, *SD*_*sTBS*_ = 3.33, *M*_*iTBS*_ = 5.40, *SD*_*iTBS*_ = 5.52).

#### State rumination contrasts

According to Mahalanobis distances (*p* < .001), there were two multivariate outliers and three participants with missing data, which were excluded for the following analysis.

Fitting the rmANOVA, we observed a significant three-way interaction of time, group, and order of stimulation conditions, *F*(3, 225) = 5.235, *p* < .01, ${\eta }_{p}^{2}$ = .065) and a significant lower-order interaction of time and order of stimulation conditions, *F*(3, 225) = 15.850, *p* < .001, ${\eta }_{p}^{2}$ = .174.

We then conducted separate rmANOVAs based on the order of the stimulation conditions. In case participants received sham stimulation first, we observed a marginally significant interaction between time and group (sham → active: *F*(3, 105) = 2.144, *p* = .099, ${\eta }_{p}^{2}$ = .058). In case participants received active stimulation first, we observed a significant interaction between time and group (active → sham: *F*(3, 120) = 4.096, *p* < .01, ${\eta }_{p}^{2}$ = .093).

**Fig. 3:**
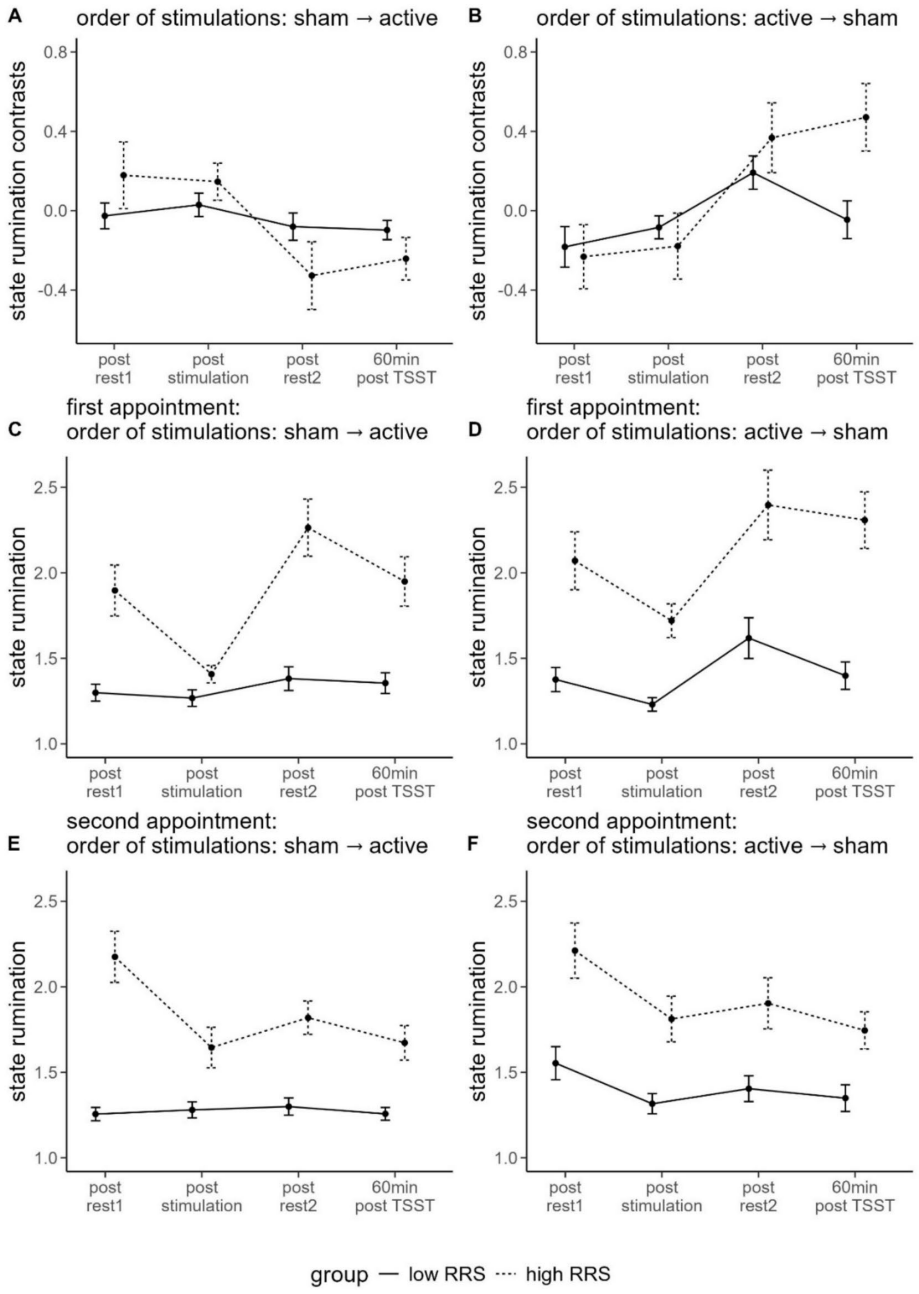
Line plot of the contrasts of state rumination ratings dependent on order of conditions (**A** = sham stimulation at the first appointment, active stimulation at the second appointment; **B** = active stimulation at the first appointment, sham stimulation at the second appointment), and line plot of raw data of state rumination ratings dependent on order of conditions and appointment (**C** = first appointment sham stimulation; **D** = first appointment active stimulation; **E** = second appointment active stimulation; **F** = second appointment sham stimulation). rest = resting-state measurement; 60 min post TSST = 60 min after the Trier Social Stress Test; RRS = Ruminative Response Scale. Error bars indicate 1 standard error of the mean

Polynomial contrasts of the interaction of time and group revealed a linear contrast but in opposite directions dependent on the order of stimulation conditions (see Figs. 3A and B). For participants having received active stimulation first, the differences between the active and passive stimulation increase and increase more in high ruminators, *F*(1, 40) = 7.235, *p* < .05, ${\eta }_{p}^{2}$ = .153. The opposite was true for participants having received sham stimulation first: Differences between the active and passive stimulation decrease and decrease more for high ruminators, *F*(1, 35) = 3.880, *p* = .057, ${\eta }_{p}^{2}$ = .100.

However, after adjusting for multiple comparisons, Benjamini-Hochberg-corrected post-hoc tests showed no significant differences between low and high ruminators at any time point.

#### State rumination raw data

After checking for multivariate outliers using Mahalanobis distances (*p* < .001), we excluded four data points and three owing to missing data, specifically two participants' data from the first and five participants' data from the second appointment, for the following analysis.

Fitting the rmANOVA on the raw data, we observed a significant interaction of time and RRS group (AP1: *F*(2.762, 220.970) = 6.559, *p* < .001, ${\eta }_{p}^{2}$ = .076; AP2: *F*(3, 231) = 8.288, *p* < .001, ${\eta }_{p}^{2}$ = .097) with quadratic polynomial contrasts, each indicating higher increases in state rumination in high ruminators (AP1: *F*(1, 80) = 4.093, *p* < .05, ${\eta }_{p}^{2}$ = .049; AP2: *F*(1, 77) = 6.870, *p* < .05, ${\eta }_{p}^{2}$ = .082). Benjamini-Hochberg-corrected pairwise comparisons yielded significant differences between low and high ruminators at any given time point (*p* < .05) (see Figs. 3C-F).

#### State rumination planned contrasts

We observed no significant differences in state rumination ratings after the second resting-state (i.e., following the stress induction) between sTBS and cTBS, neither for low nor high ruminators during appointment 1 or 2 (all *p's* > .117).

When comparing sTBS and iTBS, we observed significantly higher state rumination following sTBS (*M* = 1.40, *SD* = 0.35) compared with iTBS (*M* = 1.21, *SD* = 0.12) for low ruminators at their second appointment, *t*(27.069) = 2.305, *p* <.05, *d* = 0.661 and marginally significant differences for low ruminators at their first appointment, *t*(13.232) = −1.480,* p* = .081, *d* = −0.657, this time, however, higher state rumination following iTBS (*M* = 1.66, *SD* = 0.58) compared with sTBS (*M* = 1.38, *SD* = 0.32).

#### State rumination reliable change

Finally, we calculated Reliable Change Indices (RCI) by comparing state rumination ratings from before the stress induction (rest1) to after the stress induction (rest2), separately for each appointment and RRS group. In case of both appointments, most participants exhibited no reliable change in state rumination (see Table 4). Interestingly, about 43% of high ruminators showed reliable increases at the first appointment and only 2% at the second appointment. The same pattern was observed for low ruminators. Furthermore, whereas only three low ruminators and five high ruminators showed significant decreases in state rumination ratings due to the stress induction at the first appointment, at appointment 2, this was the case in three low and 16 high ruminators (for an illustration of RCI see supplementary material S9).

**Table 4:** Absolute and relative frequencies of reliable change

	Low ruminators	High ruminators	Test-statistic
	(*n* = 44)	(*n* = 44)	
Appointment 1:			${\upchi }^{2}$ (2) = 6.433, *p* < .05
Reliable decrease	3	5	
	(6.98%)	(11.37%)	
No reliable change	31	20	
	(72.09%)	(45.45%)	
Reliable increase	9	19	
	(20.93%)	1(43.18%)	
Appointment 2:			${\upchi }^{2}$ (2) = 11.410, *p* < .001
Reliable decrease	3	16	
	(6.81%)	(36.37%)	
No reliable change	39	27	
	(88.64%)	(61.36%)	
Reliable increase	2	1	
	(4.55%)	(2.27%)	

*Note.* Percentages refer to the relative frequencies in the corresponding subsample (low or high ruminators) and the test-statistic indicated ${\chi}^{2}$ tests comparing the distribution of RCI-categories in low and high ruminators at the respective appointment. Please note that the data of one low ruminator at the first appointment was missing

#### Negative affect contrasts

According to their Mahalanobis distances (*p* < .001), we excluded four multivariate outliers and nine owing to missing data.

Fitting the rmANOVA, we observed a significant interaction of time and order of stimulation conditions, *F*(2.750, 184.249) = 21.689, *p* < .001, ${\eta}_{p}^{2}$ = .245, and a significant main effect of order of stimulation conditions, *F*(1, 67) = 10.054, *p* < .01, ${\eta}_{p}^{2}$ = .130.

**Fig. 4:**
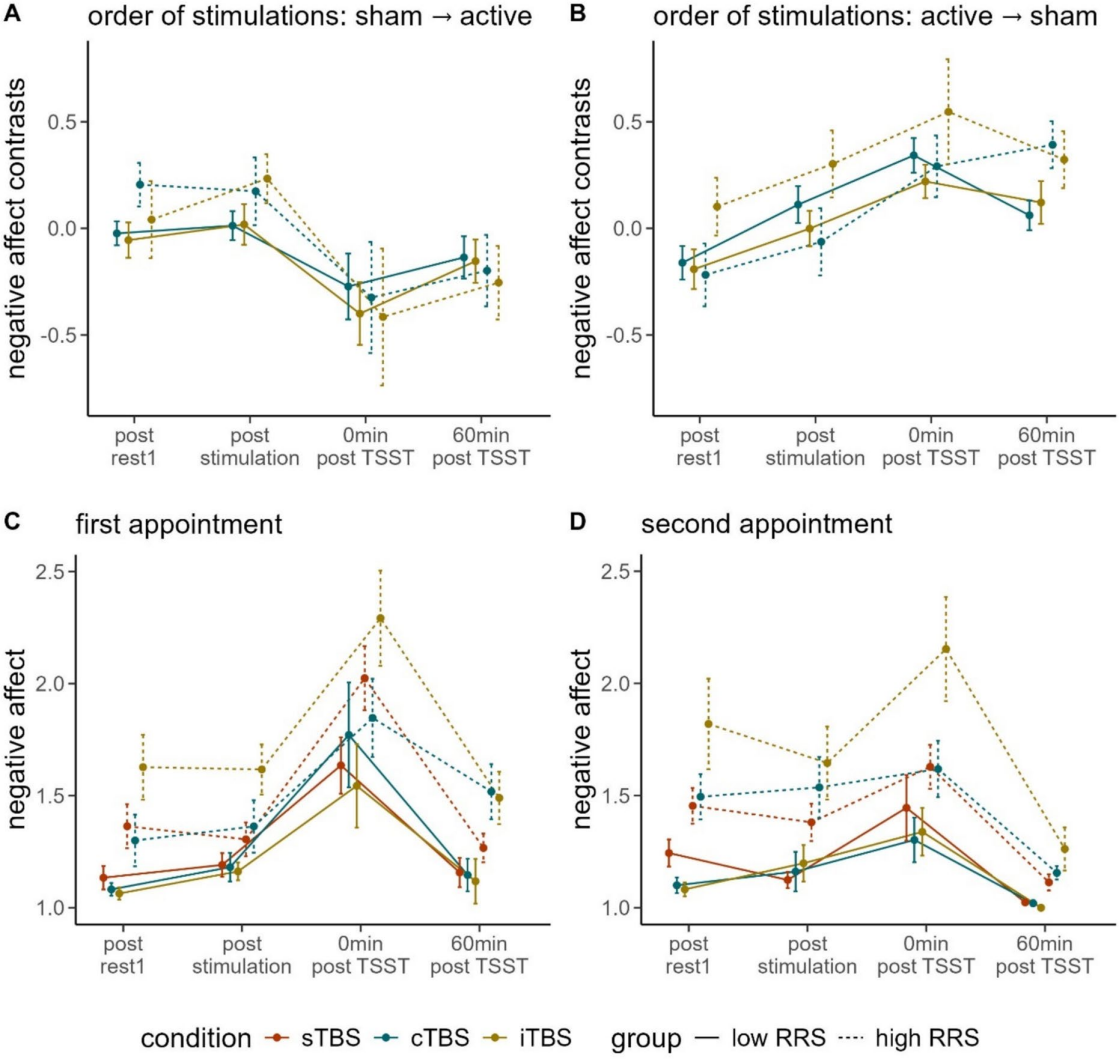
Line plot of the contrasts of negative affect ratings dependent on order of conditions (**A** = sham stimulation at the first appointment, active stimulation at the second appointment; **B** = active stimulation at the first appointment, sham stimulation at the second appointment) and line plot of raw data of negative affect ratings dependent on the appointment (**C** = first appointment; **D** = second appointment). rest = resting-state measurement; sTBS = sham Theta Burst Stimulation; cTBS = continuous Theta Burst Stimulation; iTBS = intermittent Theta Burst Stimulation; 0 min post TSST = 0 min after the Trier Social Stress Test; RRS = Ruminative Response Scale. Please note that the effect of the order of stimulation conditions is visually depicted within the plots of the different appointments (first appointment: sTBS = participants with order of stimulation conditions sham → active; iTBS and cTBS = participants with order of stimulation conditions active → sham; second appointment: vice versa). Error bars indicate 1 standard error of the mean

Consequently, we fitted the rmANOVA separately for the different stimulation conditions and observed a marginally significant three-way interaction of time, group and stimulation condition for participants having received active stimulation first, *F*(3, 99) = 2.194, *p* = .093, ${\eta}_{p}^{2}$ = .062 (see Fig. 4B). Regardless of order of stimulations, we observed a significant main effect of time (sham → active: *F*(2.401, 81.643) = 8.893, *p* < .001, ${\eta}_{p}^{2}$ = .207; active → sham: *F*(3, 99) = 19.085, *p* < .001, ${\eta }_{p}^{2}$ = .366) as well as a (marginally) significant main effect of the intercept (i.e., a significant difference between active vs. passive stimulation; sham → active: *F*(1, 34) = 3.293, *p* = .078, ${\eta}_{p}^{2}$ = .088; active → sham: *F*(1, 33) = 7.293, *p* < .05, ${\eta}_{p}^{2}$ = .181).

Further investigating the main effect of time using polynomial contrasts revealed a linear time course in case participants received sham first (see Fig. 4A), *F*(1, 34) = 11.498, *p* < .01, ${\eta}_{p}^{2}$ = .253, which was reflected by comparable negative affect prior to the TBS and more pronounced decreases in negative affect in case of sham stimulation compared with active stimulation afterwards. We further observed a quadratic time course in case participants received active stimulation first (see Fig. 4B), *F*(1, 33) = 10.656, *p* < .01, ${\eta}_{p}^{2}$ = .244, which was reflected by comparable negative affect prior to the TBS and more pronounced decreases in negative affect in case of the active stimulation compared to sham stimulation afterwards.

#### Negative affect raw data

After checking for multivariate outliers using Mahalanobis distances (*p* < .001), we excluded nine data points and nine owing to missing data, specifically ten participants' data from the first and eight participants' data from the second appointment, for the following analysis.

Investigating the raw data, we observed a significant interaction between time and RRS group, but this was only evident in the second appointment, *F*(2.268, 167.838) = 3.638, *p* < .05, ${\eta}_{p}^{2}$ = .047, with a linear polynomial contrast, *F*(1, 74) = 8.963, *p* < .01, ${\eta}_{p}^{2}$ = .108. Benjamini-Hochberg-corrected pairwise comparisons yielded significant differences between low and high ruminators at any given time point (*p* < .05).

In both rmANOVAs, the interaction of RRS group and stimulation condition yielded (marginal) significance (AP1: *F*(2, 72) = 2.386, *p* = .099, ${\eta}_{p}^{2}$ = .062; AP2: *F*(2, 76) = 3.991, *p* < .05, ${\eta}_{p}^{2}$ = .095). Benjamini-Hochberg-corrected pairwise comparisons yielded significantly lower negative affect following sTBS (*M* = 15.34, *SE* = 0.62) compared with iTBS (*M* = 18.93, *SE* = 0.88) in high ruminators at AP2 (*p* < .05).

We further observed significant (lower-order) main effects of RRS group (AP1: *F*(1, 72) = 22.213, *p* < .001, ${\eta}_{p}^{2}$ = .236; AP2: *F*(1, 74) = 34.981, *p* < .001, ${\eta }_{p}^{2}$ = .321) and time (AP1: *F*(1.887, 135.853) = 52.728, *p* < .001, ${\eta}_{p}^{2}$ = .423; AP2: *F*(2.269, 167.838) = 33.000, *p* < .001, ${\eta }_{p}^{2}$ = .308). Polynomial contrasts yielded quadratic time courses (AP1: *F*(1, 72) = 51.589, *p* < .091, ${\eta }_{p}^{2}$ = .417; AP2: *F*(1, 74) = 44.573, *p* < .001, ${\eta}_{p}^{2}$ = .376).

#### Negative affect planned contrasts

Investigating the planned contrasts of negative affect directly after the TSST, we observed significantly higher negative affect ratings following iTBS (*M* = 2.15, *SD* = 0.73) compared with sTBS (*M* = 1.63, *SD* = 0.44) but only in high ruminators at their second appointment,* t*(28) = −2.463, *p* < .05, *d* = −0.954.

### Physiological measures

#### Cortical oxygenation overall *t*-tests

**Fig. 5:**
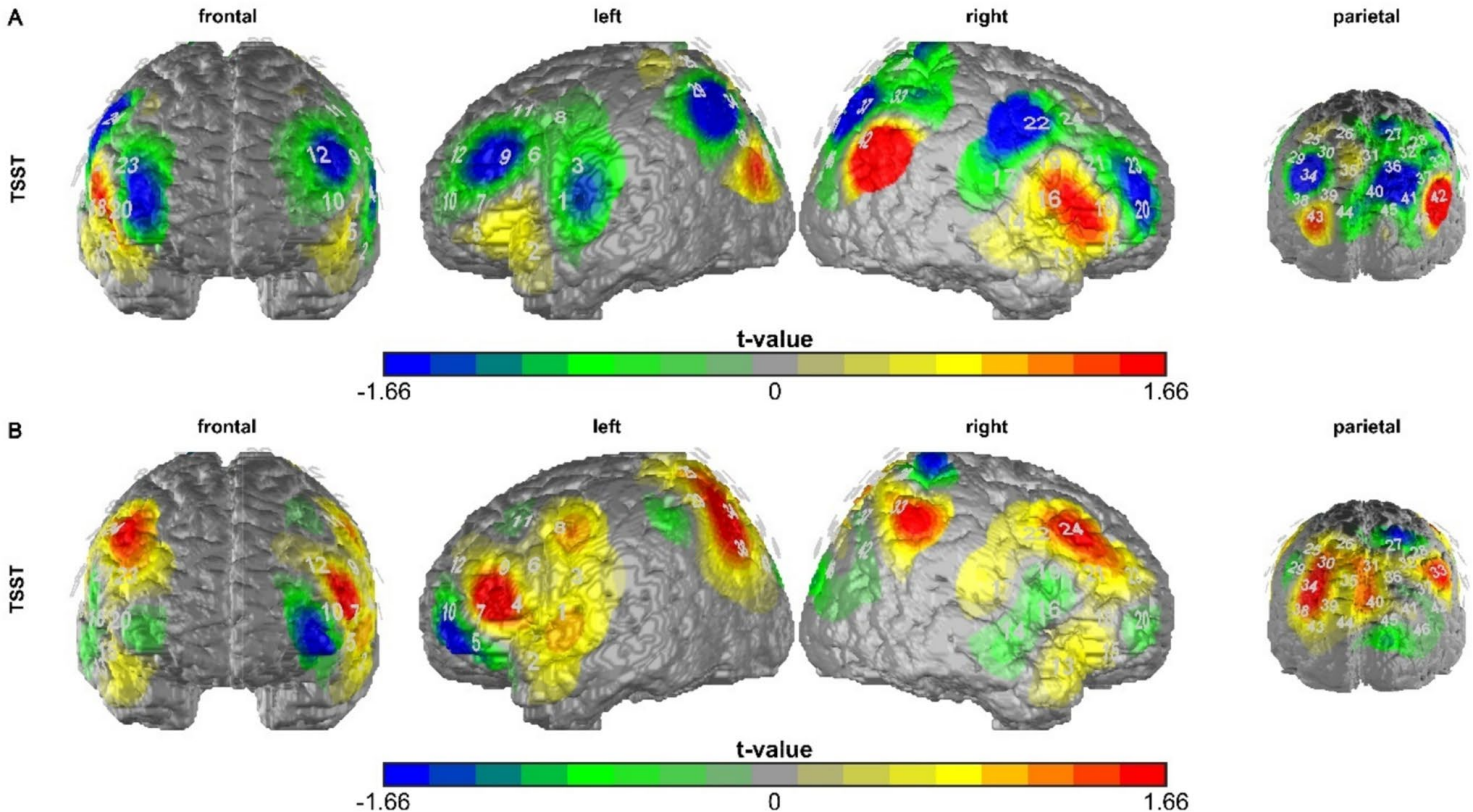
Plots of the *t*-values of the paired *t*-tests in each channel testing the active stimulation condition against the sham stimulation in each channel dependent on the stimulation condition A = cTBS, B = iTBS, TSST = arithmetic task of the TSST. The figure legend was scaled so that channels showing a significant *t*-value are displayed in blue ($\le$−1.66) or red ($\ge$ 1.66). Significant channels in the cTBS condition are channel 9 (left VLPFC), 22, 34, 36 (SAC), 41, and 42. Significant channels in the iTBS condition are channel 7 (left VLPFC), 33, and 34

We first performed paired *t*-tests to compare cortical oxygenation in each channel during the arithmetic task of the TSST, contrasting the cortical oxygenation levels after active stimulation with those following sham stimulation (active vs. sham) within subjects across all participants. Significant channels in the cTBS condition are channel 9 (left VLPFC), 22, 34, 36 (SAC), 41, and 42. Significant channels in the iTBS condition are channel 7 (left VLPFC), 33, and 34 (see Fig. 5).

#### Cortical oxygenation contrasts single channel

According to Mahalanobis distances (*p* < .001), there were no multivariate outliers but three participants with missing data.

Fitting the rmANOVA on the single channel level where the TBS was applied (channel 12/electrode position F3), we observed no significant effects (*p* > .112), only a significant quadratic polynomial contrast of the interaction of time and group, *F*(1, 77) = 4.670, *p* < .05, ${\eta }_{p}^{2}$ = .057. Descriptively, contrasts during control task 1 and the arithmetic task were comparable between low and high ruminators, while during control task 2, the low ruminators exhibited positive contrasts (i.e., higher activation during the appointment with active stimulation) compared with negative contrasts in high ruminators (i.e., higher activation during the appointment with sham stimulation).

#### Cortical oxygenation contrasts ROIs

According to Mahalanobis distances (*p* < .001), there were no multivariate outliers but three participants with missing data.

Fitting the rmMANOVA using the ROIs (left and right DLPFC, left and right VLPFC and SAC) as DVs, we observed a significant four-way interaction of time, group, stimulation condition and order of stimulation conditions, *F*(10, 302) = 3.160, Pillai's trace = 0.189, *p* < .001, ${\eta }_{p}^{2}$ = .095.

We then fitted separate rmMANOVAs dependent on the order of stimulation conditions. This resulted in a significant three-way interaction of time, group and stimulation condition but only in the case participants received active stimulation first, *F*(10, 146) = 2.733, Pillai's trace = 0.315, *p* < .01, ${\eta }_{p}^{2}$ = .158 (for brainmaps see supplementary material S10). Benjamini-Hochberg-corrected univariate tests of this three-way interaction of time, group and stimulation condition yielded significance (*p* < .05) only in the left VLPFC, *F*(2, 76) = 7.327, *p* < .01, ${\eta }_{p}^{2}$ = .162.

Polynomial contrasts of the three-way interaction of time, group and stimulation condition revealed a linear contrast in the left VLPFC, *F*(1, 38) = 13.615, *p* < .001, ${\eta }_{p}^{2}$ = .264. This was reflected by linear increases in high ruminators following cTBS and low ruminators following iTBS and decreases in high ruminators following iTBS and low ruminators following cTBS.

Benjamini-Hochberg-corrected pairwise comparisons of the three-way interaction of time, group, and stimulation condition in the left VLPFC yielded no significant differences in the contrasts (*p* < .05) between low or high ruminators at any time point, nor between the stimulation conditions at any time point. Without correction, we observed significantly lower contrasts for low ruminators (*M* = −0.43, *SD* = 0.50) compared with high ruminators after having received cTBS (*M* = 0.25, *SD* = 0.66) during the arithmetic task of the TSST, *t*(19) = −2.7, *p* < .01, *d* = −1.18.

Without correction, we further observed significantly lower contrasts during the arithmetic task of the TSST for low ruminators having received cTBS (*M* = −0.43, *SD* = 0.50) compared to low ruminators having received iTBS (*M* = 0.34, *SD* = 0.83), *t*(20) = −2.647, *p* < .01, *d* = −1.129, as well as marginally significantly higher contrasts during control task 1 for low ruminators having received cTBS (*M* = 0.25, *SD* = 0.73) compared with low ruminators having received iTBS (*M* = −0.34, *SD* = 0.68), *t*(20) = 1.963, *p* < .05, *d* = 0.837.

When we only investigated polynomial contrasts, we observed a significant three-way interaction of time, group, and stimulation condition for participants having received active stimulation first in the left DLPFC (quadratic contrast: *F*(1, 38) = 4.274, *p* < .05, ${\eta }_{p}^{2}$ = .101) as well as the right DLPFC (linear contrast: *F*(1, 38) = 5.404, *p* < .05, ${\eta }_{p}^{2}$ = .124).

In case of the left DLPFC, this effect seemed to be primarily driven by differences between low and high ruminators (positive contrasts in low and negative contrasts in high ruminators) following iTBS, as well as between the stimulation conditions (positive contrasts in case of iTBS and negative contrasts in case of cTBS) but only in low ruminators during control task 2, i.e., before the stimulation.

In the right DLPFC, on the other hand, the effect seemed to be driven by differences during the arithmetic task of the TSST, i.e., after the TBS. More specifically, we observed positive contrasts in high ruminators and negative contrasts in low ruminators following cTBS. We further observed positive contrasts during the arithmetic task following iTBS compared with negative contrasts following cTBS but only in the case of low ruminators.

Interestingly, we further observed a significant three-way interaction of time, group and stimulation condition for participants having received sham stimulation first in the right DLPFC (linear contrast: *F*(1, 39) = 7.529, *p* < .01, ${\eta }_{p}^{2}$ = .162) and the right VLPFC (linear contrast: *F*(1, 39) = 4.195, *p* < .05, ${\eta }_{p}^{2}$ = .097). In the right DLPFC during the arithmetic task of the TSST, we observed positive contrasts in low and negative contrasts in high ruminators following cTBS. In the right VLPFC during the arithmetic task of the TSST, we observed positive contrasts following cTBS and negative contrasts following iTBS but only in low ruminators.

#### Cortical oxygenation raw data ROIs

According to Mahalanobis distances (*p* < .001), there was one multivariate outlier and three participants with missing data (2 of AP1 and 1 of AP2).

Fitting the rmMANOVA separately for each appointment on the raw data, we observed a significant main effect of time for the first and second appointment (AP1: *F*(10, 314) = 5.984, Pillai's trace = 0.32, *p* < .001, ${\eta }_{p}^{2}$ = .160; AP2: *F*(10, 314) = 6.373, Pillai's trace = 0.337, *p* < .001, ${\eta }_{p}^{2}$ = .169).

Benjamini-Hochberg-corrected univariate tests revealed a significant main effect of time for all ROIs at the first appointment, and for the bilateral DLPFC, left VLPFC, and SAC at the second appointment (*p* < .05). Polynomial contrasts revealed a linear time course during AP1 in the case of the left DLPFC, *F*(1, 80) = 24.880, *p* < .001, ${\eta }_{p}^{2}$ = .237, left VLPFC, *F*(1, 80) = 9.605,* p* < .01, ${\eta }_{p}^{2}$ = .107, right DLPFC, *F*(1, 80) = 18.125, *p* < .001, ${\eta }_{p}^{2}$ = .185, and SAC, *F*(1, 80) = 57.247,* p* < .001, ${\eta }_{p}^{2}$ = .417, and a quadratic time course in case of the right VLPFC, *F*(1, 80) = 6.474, *p* < .05, ${\eta }_{p}^{2}$ = .075.

At the second appointment, we observed a linear time course in the left DLPFC, *F*(1, 80) = 17.058, *p* < .001, ${\eta }_{p}^{2}$ = .176, left VLPFC,* F*(1, 80) = 12.541, *p* < .001, ${\eta }_{p}^{2}$ = .136, SAC*, F*(1, 80) = 40.647,* p* < .001, ${\eta }_{p}^{2}$ = .337, and a quadratic time course in the right DLPFC, *F*(1, 80) = 11.548, *p* < .01, ${\eta }_{p}^{2}$ = .126.

No significant between-subjects effects were observed.

Investigating the polynomial contrasts, we further found a quadratic contrast of the three-way interaction of time, stimulation condition, and RRS group in the case of the left DLPFC at AP1, *F*(2, 80) = 3.626, *p* < .05, ${\eta }_{p}^{2}$ = .83. Descriptively, this effect was driven by higher cortical oxygenation in low ruminators compared with high ruminators during control task 2 (i.e., previous to the TBS) but comparable activation during control task 1 and the arithmetic task of the TSST.

Investigating the polynomial contrasts, we further found a linear contrast of the three-way interaction of time, stimulation condition, and RRS group in the case of the right DLPFC at AP2, *F*(2, 80) = 4.582, *p* < .05, ${\eta }_{p}^{2}$ = .103. Descriptively, this effect seemed to be driven by differences between low and high ruminators during control task 1 having received cTBS (higher cortical oxygenation in high ruminators) (i.e., previous to the TBS).

Lastly, we observed a significant linear contrast of the interaction of time and RRS group in case of the left VLPFC, *F*(1, 80) = 9.014, *p* < .01, ${\eta }_{p}^{2}$ = .101, and right VLPFC at AP2, *F*(1, 80) = 4.362, *p* < .05, ${\eta }_{p}^{2}$ = .052. Descriptively, the interaction of time and RRS group in the right VLPFC was reflected by higher cortical oxygenation in case of the high ruminators during control task 1, comparable cortical oxygenation during control task 2 and higher cortical oxygenation during the arithmetic task of the TSST in case of low ruminators. In case of the left VLPFC, this interaction was primarily driven by significantly higher cortical oxygenation in high ruminators during control task 1.

#### Cortical oxygenation planned contrasts

**Fig. 6:**
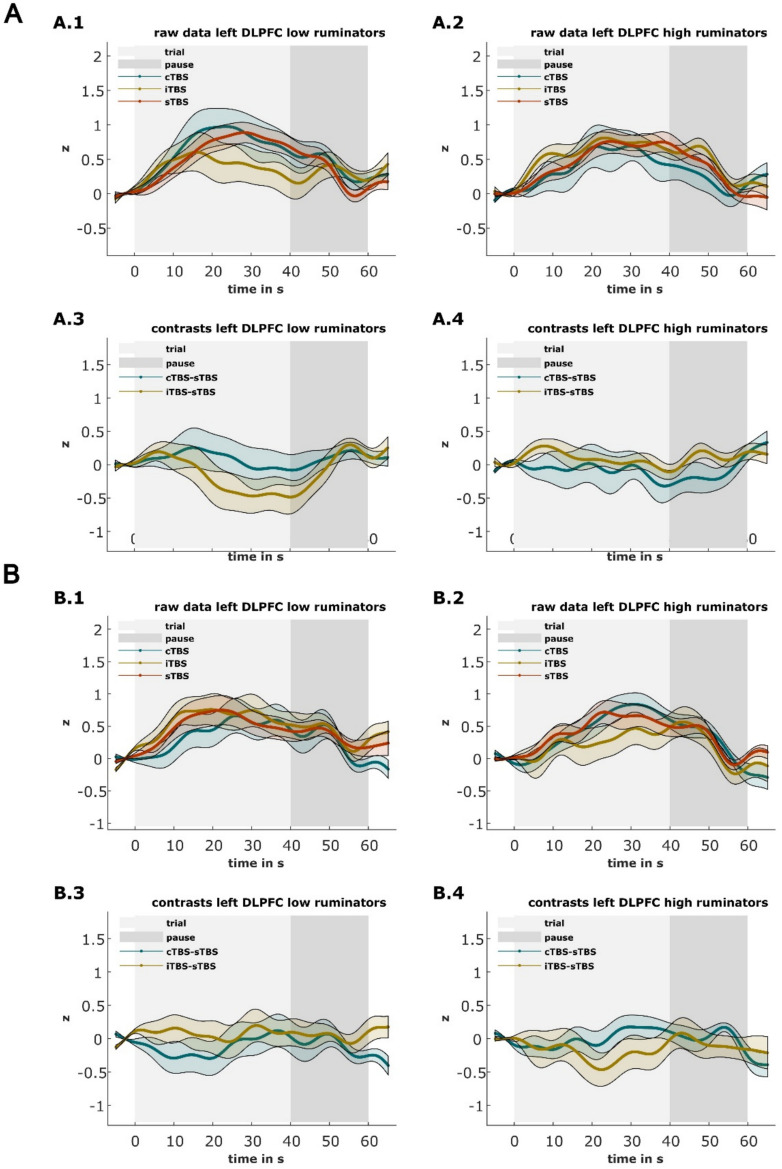
The z-standardized hemodynamic responses during the arithmetic task of the TSST in the left DLPFC in low and high ruminators depending on the TBS-condition at the first appointment (**A**) and the second appointment (**B**). A.1, A.2, B.1, and B.2 illustrate the raw data. A.3, A.4, B.3, and B.4 illustrate the contrasts (cTBS minus sTBS and iTBS minus sTBS). The light shading marks the 40 s trial and the dark shading the 20 s pause to allow the hemodynamic response to recover. Shadings around the hemodynamic curves reflect standard errors of the mean. The baseline includes the 5 s before each trial; 0 s on the x-axis marks the beginning of the trial. See supplementary material S11 for the time series of the other ROIs

Lastly, we examined potential differences in cortical oxygenation in the left DLPFC during the arithmetic task of the TSST (i.e., following TBS) based on the stimulation condition, separately for low and high ruminators, and for each appointment. These planned contrasts yielded no significant differences (all *p's* > .157) (for an illustration of the time series in the left DLPFC; see Fig. 6).

#### Exploratory analysis: Impact of expectancy effects

When we investigated the impact of expectancy effects, we observed no significant main effects or interaction with time in the case of subjective stress or cortical oxygenation. For state rumination, there was a significant interaction with time at the first appointment, *F(*2.851, 205.272) = 3.129, *p* < .05, ${\eta }_{p}^{2}$ = .042. In case of negative affect, we observed a marginally significant interaction of expectancy effects and time at the first appointment, *F*(1.981, 128.763) = 2.695, *p* = .072, ${\eta }_{p}^{2}$ = .04, and a significant main effect at the second appointment, *F*(1, 75) = 4.757, *p* < .05, ${\eta }_{p}^{2}$ = .06.

## Discussion

This study was designed to investigate the impact of modulating left DLPFC activity on the stress-rumination link using sham-controlled TBS. For this purpose, a total of 88 healthy participants comprising 44 low and 44 high trait ruminators underwent a neurostimulation prior to a social stress induction using the Trier Social Stress Test. At one appointment, participants either received cTBS (i.e., inhibitory) or iTBS (i.e., excitatory), and the other one 5 weeks apart sTBS (sham), with the order randomized and balanced between subjects. Throughout the experiment, we assessed cortical oxygenation using fNIRS, heart rate, and psychological measures, including subjective stress, state rumination, and positive and negative affect.

First, all manipulation checks concerning the blinding, stress induction, and stratification were successfully met: All study subsamples were comparable regarding their respective distribution of demographic and clinical variables. Furthermore, the blinding of the TBS seemed to be successful as participants were unable to identify real from sham stimulation. Moreover, the stress induction using the TSST was successful as we observed significant increases in subjective stress, heart rates, and negative affect due to the stress induction, at both the first as well as second appointments, replicating findings of previous studies proposing the TSST as a highly ecologically valid stressor and the “gold standard” for the investigation of the psychophysiological stress response (Allen et al., 2014).

When examining the effects of TBS on the dependent variables, we observed a highly complex interplay between time-dependent changes, group differences between low and high ruminators, TBS effects, habituation effects, and the order in which stimulation conditions were administered (i.e., whether participants received active stimulation in their first session and sham in their second, or vice versa). We acknowledge that the analyses were relatively complex and that including stimulation order is not common practice. However, given the strong habituation effects observed across nearly all dependent variables, we deemed this factor crucial. Stress responses were most pronounced during the first exposure, likely due to novelty effects, potentially masking small TBS effects. Initial rmANOVAs without stimulation order yielded predominantly null results, leading us to include this factor to better understand how habituation interacts with stimulation effects on stress reactivity.

The importance of accounting for stimulation order becomes particularly evident in the analysis of subjective stress responses. For instance, we found a significant three-way interaction of time, group, and stimulation condition but only in the sham → active sequence. This interaction was primarily driven by high ruminators in the iTBS-arm, who reported higher stress during stress induction when they received sham stimulation at the first session, compared with high ruminators in the cTBS-arm, who reported lower stress when receiving sham at the first session (i.e., sham with the duration of cTBS). At the second appointment, overall stress responses were lower; the iTBS group showed a more pronounced reduction, while the cTBS group exhibited non-significant changes.

Planned contrasts comparing post-stress stress levels revealed a significant difference between iTBS and sTBS, although in an unexpected direction: stress levels were higher following iTBS compared with sTBS, particularly at the first appointment and most pronounced in high ruminators. These findings align intuitively with the results of the negative affect analysis. While overall analyses did not reveal significant TBS effects, planned contrasts comparing post-stress negative affect indicated higher negative affect following iTBS compared with sTBS, again in high ruminators but only at the second appointment. These findings suggest that the effects are highly complex, requiring large sample sizes to detect small effects within such intricate experimental designs.

Similarly, for state rumination, overall analyses did not indicate a significant TBS effect, but planned contrasts at the second appointment revealed a pattern consistent with our hypotheses: low ruminators in the sTBS condition reported higher state rumination post-stress (rest2) compared with those in the iTBS condition, aligning with the expected excitatory effects of iTBS. Please note, however, that increases in state rumination were generally low and only in case of the first appointment strong increases in state rumination were found in the high ruminators, reflected by reliable change indices.

We did not find any impact of the stimulation on heart rate, positive affect, and performance during the TSST.

The major advantage of the current study setup compared to the previous studies on the impact of TBS on stress-reactive rumination (De Smet et al., 2024; De Witte et al., 2020) is the additional assessment of brain activation prior to and after the stimulation. Regarding the results of cortical oxygenation, we could replicate the effect of a stress-induced increase in cortical oxygenation in prefrontal areas that might be interpreted as a successful stress induction owing to the need for increased resources. We then initially focused on the single fNIRS channel corresponding to electrode position F3, where TBS was applied. This analysis did not reveal any significant effects of TBS. Expanding the analysis by using the ROI, including the mean signal from the three channels covering the left DLPFC in the repeated-measures MANOVA, we found no overall significant differences in the left DLPFC between stimulation conditions during the TSST — only differences between low and high ruminators previous to the TBS. Additionally, we observed a significant influence of stimulation order: Specifically, a significant three-way interaction of time, group, and stimulation condition emerged in the left VLPFC but only in participants who received active stimulation at their first appointment and sham at their second. However, given the complexity of this analysis and the small effect sizes, the following results reached significance only without correction for multiple comparisons. Interestingly, in the left VLPFC, we found stimulation-related differences in line with our hypotheses but exclusively in low ruminators and only when stimulation was administered in the active → sham order. More precisely, we observed negative contrasts in cortical oxygenation following cTBS (i.e., greater activation after sham compared with active stimulation, indicating an inhibitory effect of cTBS) and positive contrasts following iTBS (i.e., lower activation after sham compared with active stimulation, suggesting an excitatory effect of iTBS). A similar pattern was found in the right DLPFC when polynomial contrasts were examined. Interestingly, the right VLPFC exhibited the opposite pattern in the sham → active stimulation order — meaning that in low ruminators, cTBS had an excitatory effect, whereas iTBS had an inhibitory effect during the arithmetic task of the TSST.

What is particularly interesting is that we did not observe the well-documented prefrontal hypoactivation under stress in the left DLPFC, which has been repeatedly found for patients with depression (Pizzagalli & Roberts, 2022) and has also been observed to be associated with trait rumination (Int-Veen et al., 2023), which is why we expected to replicate these findings in high ruminators at least in the sTBS condition.

One explanation for finding significant effects of the stimulation in some but not all measures may be differences in noise and effect sizes in the corresponding measures. Because of the distinct underlying physiological mechanisms and measurement methods, it seems reasonable to assume diverse measurement errors and reliability. Please keep in mind that these might also be differently affected by the stimulation itself, which is why ultimately a huge variance in actual data, as well as errors, might arise and is not sufficiently explained by the current analysis.

There are several possible interpretations of these inconclusive TBS effects beyond the already mentioned different measurement errors of physiological, neurophysiological, and behavioral data. On the one hand, these findings may result from a potential diminishing of the TBS effect in the case of the very pronounced hemodynamic response induced by using an experimental stress induction like the TSST. This is partly due to the TSST being a very potent stressor evoking strong subjective and physiological responses (Allen et al., 2014) and on the other hand due to the block design of the study (the control tasks as well as the arithmetic task of the TSST) where multiple hemodynamic response functions linearly add up.

Another interpretation of the absence or inconsistency of TBS effects could be the timing of the stimulation within the experimental procedure. While the duration of TBS effects seems to differ systematically depending on the protocol (Lowe et al., 2018), we aimed to minimize the time between the end of the TBS and the stress induction. This is also why we did not perform the stimulation before the control tasks. As a consequence, approximately 11 min (*SD* = 6 min) passed between the end of the TBS and the beginning of the TSST. With a duration of approximately 15 min of the TSST, a 7 min resting-state measurement and instructions about the further procedure, the assessment of stress-reactive state rumination was approximately 50 min (*SD* = 8 min) after the end of the TBS. This rather long time passing might also have an impact on the magnitude of the TBS effects and the potential vanishing of significance. Speaking of the timing of the TBS, one further limitation of the current study setup is that we were not able to measure cortical oxygenation during the stimulation itself (Kozel et al., 2009). It should be noted that the timing of the fNIRS measurement and the functioning of the continuous-wave fNIRS device may have additionally contributed to the inadequate representation of the TBS effects; only relative (as opposed to absolute) changes in hemoglobin concentration over time, relative to a baseline measurement taken at the beginning of each measurement segment, are recorded. It is likely that during the fNIRS measurement conducted during stress induction, the baseline was influenced by the preceding TBS, which may have artificially reset the various TBS conditions to zero. Therefore, only differences between the TBS conditions can become significant if brain activity during the TSST continues to be inhibited or increased.

Recent studies with special hardware are able to assess fNIRS simultaneously with the stimulation, which offers the chance for online feedback and as a consequence new insights regarding TBS-induced changes in cortical oxygenation.

Finally, another important point of discussion is coil positioning, which may not have been optimal when using the electrode position F3 instead of a neuronavigation system. However, it is important to note that structural fMRI data were not available for this study, limiting the feasibility of this approach. To account for potential small deviations in coil placement, we reanalyzed the data by using both the single-channel covering F3 and a merged signal from the three channels of the left DLPFC. These additional analyses did not yield any systematically different findings. Furthermore, the stimulation location and target of the TBS itself might also be suboptimal as suggested by a recent meta-analysis (Moses et al., 2023) on the effects of neuromodulation of the cognitive and emotional stress response in healthy individuals. Following this meta-analysis, the predominant share of studies found no significant effects of NIBS on emotional reactivity and salivary cortisol following a stress induction. Furthermore, although “all of the studies used a between-subjects design, there were no other common design features and the wide-ranging approaches prohibit clear conclusions from these studies about potential optimal targets for modulating the effects of stress on working memory.” (page 17, Moses et al., 2023). Surely, the field of NIBS is still evolving and further studies are needed to draw a more conclusive pattern of findings; however, first evidence suggests the VLPFC as a key structure in cognitive reappraisal circuits “may be the best-supported target to affect stress modulation of emotional responses” (page 16, Moses et al., 2023). We plan to conduct a study with the same study setup but this time using the right VLPFC as a target for TBS (Int-Veen, Eisenlohr, et al., n.d a). With the knowledge of these findings, eventually, we will be able to gain more trust in any of the aforementioned interpretations of the current results.

In an exploratory analysis, we further investigated the impact of expectancy effects. We first analyzed whether, dependent on the stimulation protocol, participants believed that the stimulation had an impact on their performance (better or worse) or not. Results indicated no significant effect of the stimulation condition: Generally, irrespective of the TBS, approximately half of all participants thought that the TBS had no impact on their performance, whereas approximately 20% said they thought the stimulation made them worse and 20–30% thought the stimulation made them better. We then investigated whether participants believed that the stimulation made them better or worse at the task and whether this predicted the outcome variables. While we did not find a significant impact of expectancy effects on subjective stress and cortical oxygenation. State rumination and negative affect seemed to be somewhat influenced by the anticipated effects of TBS on performance during the TSST. Please note that we abstained from a further exploratory analysis, because expectations were not uniformly distributed, were not manipulated, and the sample size would be too small for conclusive interpretation when the analysis is performed separately for each stimulation condition, RRS group, and appointment. Nevertheless, the aforementioned results hint at a potentially important association of external or internal attribution and subjective self-assessments, which should be considered in future TBS research.

Another point to consider in the context of potential impacts on expectancy effects is that we did observe a significant impact of the order of stimulation conditions (active → sham vs. sham → active) in almost all variables. The repeated measures design is likely susceptible to expectancy effects; however, we chose this setup to effectively reduce the impact of between-person error and increase statistical power to detect smaller effect sizes.

We further noted that with the study design, state rumination increases were generally low after the (TBS and) TSST (AP1: *F*(1, 85) = 16.188, *p* < .001, ${\eta }_{p}^{2}$ = .160; AP2:* F*(1, 82) = 15.855, *p* < .001, ${\eta }_{p}^{2}$ = .162) compared with previous studies with similar study setups but no TBS, which may underscore the role of expectancy effects especially in the development of rumination (see supplementary material S12 for a comparison of state rumination in the current and previous studies). However, the actual underlying causal mechanism remains unclear and has to be evaluated in future studies.

One last limitation should be noted concerning the interpretation of the current findings. After having recruited approximately half of all participants, we noticed substantial changes in the online assessment of the RRS, which was used to recruit a stratified sample of low and high trait ruminators, compared with their respective RRS scores of the paper-pencil version at the first appointment. Because of the suspected careless responses, we changed the recruitment procedure by including a second online assessment of the RRS as well as specific rules for excluding participants with changes in RRS scores (for details and analysis of predictors of changes in RRS scores, see Int-Veen et al., 2024). These results call into question whether the recruited sample truly exhibits the assumed levels of trait rumination. Potentially, this could explain why no pronounced prefrontal hypoactivation was found in high ruminators (Int-Veen et al., 2023; Rosenbaum, Hilsendegen et al., 2018a, Rosenbaum, Thomas et al., 2018b, Rosenbaum et al., 2021).

In future research, it will be essential to delve deeper into the complex effects of TBS to understand the underlying mechanisms. Specifically, the impact of the order of stimulation conditions and the strong effects of habituation on psychological variables need further investigation.

## Supplementary Information

Below is the link to the electronic supplementary material.

Supplementary file1 (PDF 2.72 MB)

## Data Availability

Data are available from the first and last authors upon reasonable request.

## Footnotes

1 Please note that we observed these issues only halfway through recruiting. Consequently, thereafter participants had to complete another online RRS 1 week prior to their first appointment where we again checked whether participants completed the RRS as a trait measure, which should remain moderately stable, and excluded participants even at their first appointment in case their RRS score deviated substantially from the initial online screening. For an analysis of this issue, we refer to Int-Veen et al. (2024).

## Code availability

Code is available from the first and last authors upon reasonable request.

## References

[CR1] Allen, A. P., Kennedy, P. J., Cryan, J. F., Dinan, T. G., & Clarke, G. (2014). Biological and psychological markers of stress in humans: Focus on the Trier Social Stress Test. *Neuroscience & Biobehavioral Reviews,**38*, 94–124. 10.1016/j.neubiorev.2013.11.005 24239854 10.1016/j.neubiorev.2013.11.005

[CR2] Allen, A. P., Kennedy, P. J., Dockray, S., Cryan, J. F., Dinan, T. G., & Clarke, G. (2017). The Trier Social Stress Test: Principles and practice. *Neurobiology of Stress,**6*, 113–126. 10.1016/j.ynstr.2016.11.001 28229114 10.1016/j.ynstr.2016.11.001 PMC5314443

[CR3] Baeken, C., Vanderhasselt, M.-A., Remue, J., Rossi, V., Schiettecatte, J., Anckaert, E., & De Raedt, R. (2014). One left dorsolateral prefrontal cortical HF-rTMS session attenuates HPA-system sensitivity to critical feedback in healthy females. *Neuropsychologia,**57*, 112–121. 24593899 10.1016/j.neuropsychologia.2014.02.019

[CR4] Beck, A. T., Steer, R. A., Brown, G. K., et al. (1996). *Beck depression inventory*. APA PsycTests.

[CR5] Berman, M. G., Peltier, S., Nee, D. E., Kross, E., Deldin, P. J., & Jonides, J. (2011). Depression, rumination and the default network. *Social Cognitive and Affective Neuroscience,**6*(5), 548–555. 10.1093/scan/nsq080 20855296 10.1093/scan/nsq080 PMC3190207

[CR6] Blampied, N. M. (2022). Reliable change and the reliable change index: Still useful after all these years? *The Cognitive Behaviour Therapist,**15*, e50.

[CR7] Cooney, R. E., Joormann, J., Eugène, F., Dennis, E. L., & Gotlib, I. H. (2010). Neural correlates of rumination in depression. *Cognitive, Affective & Behavioral Neuroscience,**10*(4), 470–478. 10.3758/CABN.10.4.470 10.3758/CABN.10.4.470 PMC4476645 21098808

[CR8] Cui, X., Bray, S., & Reiss, A. L. (2010). Functional Near Infrared Spectroscopy (NIRS) signal improvement based on negative correlation between oxygenated and deoxygenated hemoglobin dynamics. *NeuroImage,**49*(4), 3039. 10.1016/j.neuroimage.2009.11.050 19945536 10.1016/j.neuroimage.2009.11.050 PMC2818571

[CR9] Curtin, A., Tong, S., Sun, J., Wang, J., Onaral, B., & Ayaz, H. (2019). A systematic review of integrated functional near-infrared spectroscopy (fNIRS) and transcranial magnetic stimulation (TMS) studies. *Frontiers in Neuroscience,**13*, 84. 10.3389/fnins.2019.00084 30872985 10.3389/fnins.2019.00084 PMC6403189

[CR10] De Smet, S., Baeken, C., De Raedt, R., Pulopulos, M. M., Razza, L. B., Van Damme, S., De Witte, S., Brunoni, A. R., & Vanderhasselt, M.-A. (2021). Effects of combined theta burst stimulation and transcranial direct current stimulation of the dorsolateral prefrontal cortex on stress. *Clinical Neurophysiology,**132*(5), 1116–1125. 33773176 10.1016/j.clinph.2021.01.025

[CR11] De Smet, S., Int-Veen, I., Vanhollebeke, G., Pulopulos, M. M., Barth, B., Pasche, S., Baeken, C., Nuerk, H.-C., Plewnia, C., Nieratschker, V., et al. (2024). Trait-dependent effects of theta burst stimulation after psychosocial stress: A sham-controlled study in healthy individuals. *Clinical Neurophysiology,**162*, 235–247. 38556367 10.1016/j.clinph.2024.03.016

[CR12] De Witte, S., Baeken, C., Pulopulos, M. M., Josephy, H., Schiettecatte, J., Anckaert, E., De Raedt, R., & Vanderhasselt, M.-A. (2020). The effect of neurostimulation applied to the left dorsolateral prefrontal cortex on post-stress adaptation as a function of depressive brooding. *Progress in Neuro-Psychopharmacology and Biological Psychiatry,**96*, 109687. 10.1016/j.pnpbp.2019.109687 31356848 10.1016/j.pnpbp.2019.109687

[CR13] Deng, Z.-D., Lisanby, S. H., & Peterchev, A. V. (2013). Electric field depth–focality tradeoff in transcranial magnetic stimulation: Simulation comparison of 50 coil designs. *Brain Stimulation,**6*(1), 1–13. 22483681 10.1016/j.brs.2012.02.005 PMC3568257

[CR14] Era, V., Carnevali, L., Thayer, J. F., Candidi, M., & Ottaviani, C. (2021). Dissociating cognitive, behavioral and physiological stress-related responses through dorsolateral prefrontal cortex inhibition. *Psychoneuroendocrinology,**124*, 105070. 33310375 10.1016/j.psyneuen.2020.105070

[CR15] Fishburn, F. A., Ludlum, R. S., Vaidya, C. J., & Medvedev, A. V. (2019). Temporal Derivative Distribution Repair (TDDR): A motion correction method for fNIRS. *NeuroImage,**184*, 171–179. 10.1016/j.neuroimage.2018.09.025 30217544 10.1016/j.neuroimage.2018.09.025 PMC6230489

[CR16] Gaynes, B. N., Lloyd, S. W., Lux, L., Gartlehner, G., Hansen, R. A., Brode, S., Jonas, D. E., Evans, T. S., Viswanathan, M., & Lohr, K. N. (2014). Repetitive transcranial magnetic stimulation for treatment-resistant depression: A systematic review and meta-analysis. *The Journal of Clinical Psychiatry,**75*(5), 29758. 10.4088/JCP.13r08815 24922485

[CR17] Hamilton, J. P., Furman, D. J., Chang, C., Thomason, M. E., Dennis, E., & Gotlib, I. H. (2011). Default-Mode and Task-Positive Network Activity in Major Depressive Disorder: Implications for Adaptive and Maladaptive Rumination. *Biological Psychiatry,**70*(4), 327–333. 10.1016/j.biopsych.2011.02.003 21459364 10.1016/j.biopsych.2011.02.003 PMC3144981

[CR18] Hamilton, J. P., Farmer, M., Fogelman, P., & Gotlib, I. H. (2015). Depressive Rumination, the Default-Mode Network, and the Dark Matter of Clinical Neuroscience. *Biological Psychiatry,**78*(4), 224–230. 10.1016/j.biopsych.2015.02.020 25861700 10.1016/j.biopsych.2015.02.020 PMC4524294

[CR19] Hautzinger, M., Keller, F., & Kühner, C. (2009). *Beck—Depressions—Inventar (BDI-II) Deutsche Ausgabe. Harcourt Test Services GmbH* (2nd ed.). Pearson Assessment.

[CR20] Henze, G.-I., Zänkert, S., Urschler, D. F., Hiltl, T. J., Kudielka, B. M., Pruessner, J. C., & Wüst, S. (2017). Testing the ecological validity of the trier social stress test: Association with real-life exam stress. *Psychoneuroendocrinology,**75*, 52–55. 10.1016/j.psyneuen.2016.10.002 27771565 10.1016/j.psyneuen.2016.10.002

[CR21] Henze, G.-I., Rosenbaum, D., Bärtl, C., Laicher, H., Konzok, J., Kudielka, B. M., Fallgatter, A. J., Wüst, S., Ehlis, A.-C., & Kreuzpointner, L. (2023). Comparing two psychosocial stress paradigms for imaging environments–ScanSTRESS and fNIRS-TSST: Correlation structures between stress responses. *Behavioural Brain Research,**436*, 114080. 36030907 10.1016/j.bbr.2022.114080

[CR22] Huang, Y.-Z., Edwards, M. J., Rounis, E., Bhatia, K. P., & Rothwell, J. C. (2005). Theta burst stimulation of the human motor cortex. *Neuron,**45*(2), 201–206. 10.1016/j.neuron.2004.12.033 15664172 10.1016/j.neuron.2004.12.033

[CR23] Huang, Y.-Z., Rothwell, J. C., Chen, R.-S., Lu, C.-S., & Chuang, W.-L. (2011). The theoretical model of theta burst form of repetitive transcranial magnetic stimulation. *Clinical Neurophysiology,**122*(5), 1011–1018. 20869307 10.1016/j.clinph.2010.08.016 PMC3046904

[CR24] IBM Corp. (2021). *IBM SPSs statistics for windows* (Version 28.0). IBM Corp.

[CR25] Int-Veen, I., Fallgatter, A. J., Ehlis, A.-C., & Rosenbaum, D. (2023). Prefrontal hypoactivation induced via social stress is more strongly associated with state rumination than depressive symptomatology. *Scientific Reports,**13*(1), 15147. 37704652 10.1038/s41598-023-41403-y PMC10499935

[CR26] Int-Veen, I., Eisenlohr, C., Täglich, R., Schopp, B., Nuerk, H.-C., Plewnia, C., De Smet, S., Vanderhasselt, M.-A., Kroczek, A., Barth, B., Fallgatter, A. J., Ehlis, A.-C., & Rosenbaum, D. (n.d a). *Strong habituation in the context of stress and rumination: Insights from repeated Theta Burst Stimulation of the right Ventrolateral Prefrontal Cortex*. 10.1038/s41598-025-15099-1 PMC12375055 40849567

[CR27] Int-Veen, I., Laicher, H., Ehlis, A.-C., Fallgatter, A. J., & Rosenbaum, D. (n.d b). *Measuring state rumination: Development and psychometric evaluation of the stress-reactive state rumination questionnaire (SRSRQ)*.

[CR28] Int-Veen, I., Ehlis, A.-C., Fallgatter, A. J., & Rosenbaum, D. (2024). On assessing trait rumination using the Ruminative Response Scale. *Frontiers in Psychology,**15*, 1368390. 38899126 10.3389/fpsyg.2024.1368390 PMC11186473

[CR29] Jacob, Y., Morris, L. S., Huang, K.-H., Schneider, M., Rutter, S., Verma, G., Murrough, J. W., & Balchandani, P. (2020). Neural correlates of rumination in major depressive disorder: A brain network analysis. *NeuroImage: Clinical,**25*, 102142. 10.1016/j.nicl.2019.102142 31901654 10.1016/j.nicl.2019.102142 PMC6940660

[CR30] Jasper, H. H. (1958). Report of the committee on methods of clinical examination in electroencephalography. *Electroencephalography and Clinical Neurophysiology,**10*(2), 370–375. 10.1016/0013-4694(58)90053-1

[CR31] Jones, N. P., Fournier, J. C., & Stone, L. B. (2017). Neural correlates of autobiographical problem-solving deficits associated with rumination in depression. *Journal of Affective Disorders,**218*, 210–216. 10.1016/j.jad.2017.04.069 28477499 10.1016/j.jad.2017.04.069 PMC5505343

[CR32] Kirschbaum, C., Pirke, K.-M., & Hellhammer, D. H. (1993). The ‘Trier Social Stress Test’ – A Tool for investigating psychobiological stress responses in a laboratory setting. *Neuropsychobiology,**28*(1–2), 76–81. 10.1159/000119004 8255414 10.1159/000119004

[CR33] Kozel, F. A., Tian, F., Dhamne, S., Croarkin, P. E., McClintock, S. M., Elliott, A., Mapes, K. S., Husain, M. M., & Liu, H. (2009). Using simultaneous repetitive transcranial magnetic stimulation/functional near infrared spectroscopy (rTMS/fNIRS) to measure brain activation and connectivity. *Neuroimage,**47*(4), 1177–1184. 19446635 10.1016/j.neuroimage.2009.05.016 PMC2728000

[CR34] Kudielka, B. M., Hellhammer, D. H., & Kirschbaum, C. (2007). Ten years of research with the trier social stress test-revisited. *Integrating biological and psychological explanations of social behavior* (pp. 56–83). The Guilford Press.

[CR35] Kühn, S., Vanderhasselt, M.-A., De Raedt, R., & Gallinat, J. (2014). The neural basis of unwanted thoughts during resting state. *Social Cognitive and Affective Neuroscience,**9*(9), 1320–1324. 23929943 10.1093/scan/nst117 PMC4158367

[CR36] Lowe, C. J., Manocchio, F., Safati, A. B., & Hall, P. A. (2018). The effects of theta burst stimulation (TBS) targeting the prefrontal cortex on executive functioning: A systematic review and meta-analysis. *Neuropsychologia,**111*, 344–359. 29438672 10.1016/j.neuropsychologia.2018.02.004

[CR37] Mandell, D., Siegle, G. J., Shutt, L., Feldmiller, J., & Thase, M. E. (2014). Neural substrates of trait ruminations in depression. *Journal of Abnormal Psychology,**123*(1), 35–48. 10.1037/a0035834 24661157 10.1037/a0035834 PMC4128503

[CR38] MATLAB. (2024). *Version R2024a*. The MathWorks Inc.

[CR39] Moses, T. E., Gray, E., Mischel, N., & Greenwald, M. K. (2023). Effects of neuromodulation on cognitive and emotional responses to psychosocial stressors in healthy humans. *Neurobiology of Stress,**22*, 100515. 36691646 10.1016/j.ynstr.2023.100515 PMC9860364

[CR40] Murphy, E. R., Barch, D. M., Pagliaccio, D., Luby, J. L., & Belden, A. C. (2016). Functional connectivity of the amygdala and subgenual cingulate during cognitive reappraisal of emotions in children with MDD history is associated with rumination. *Developmental Cognitive Neuroscience,**18*, 89–100. 10.1016/j.dcn.2015.11.003 26746624 10.1016/j.dcn.2015.11.003 PMC4834229

[CR41] Nejad, A. B., Fossati, P., & Lemogne, C. (2013). Self-Referential Processing, Rumination, and Cortical Midline Structures in Major Depression. *Frontiers in Human Neuroscience*, *7*. 10.3389/fnhum.2013.00666 10.3389/fnhum.2013.00666 PMC3794427 24124416

[CR42] Nolen-Hoeksema, S., Wisco, B. E., & Lyubomirsky, S. (2008). Rethinking rumination. *Perspectives on Psychological Science,**3*(5), 400–424. 10.1111/j.1745-6924.2008.00088.x 26158958 10.1111/j.1745-6924.2008.00088.x

[CR43] Oberman, L. (2014). Repetitive transcranial magnetic stimulation (rTMS) protocols. In A. Rotenberg, J. C. Horvath, & A. Pascual-Leone (Eds.), *Transcranial magnetic stimulation* (pp. 129–139). Springer.

[CR44] Okamoto, M., & Dan, I. (2005). Automated cortical projection of head-surface locations for transcranial functional brain mapping. *Neuroimage,**26*(1), 18–28. 15862201 10.1016/j.neuroimage.2005.01.018

[CR45] Okamoto, M., Dan, H., Sakamoto, K., Takeo, K., Shimizu, K., Kohno, S., Oda, I., Isobe, S., Suzuki, T., Kohyama, K., et al. (2004). Three-dimensional probabilistic anatomical cranio-cerebral correlation via the international 10–20 system oriented for transcranial functional brain mapping. *Neuroimage,**21*(1), 99–111. 14741647 10.1016/j.neuroimage.2003.08.026

[CR46] Ottaviani, C., Thayer, J. F., Verkuil, B., Lonigro, A., Medea, B., Couyoumdjian, A., & Brosschot, J. F. (2016). Physiological concomitants of perseverative cognition: A systematic review and meta-analysis. *Psychological Bulletin,**142*(3), 231–259. 10.1037/bul0000036 26689087 10.1037/bul0000036

[CR47] Philippi, C. L., Cornejo, M. D., Frost, C. P., Walsh, E. C., Hoks, R. M., Birn, R., & Abercrombie, H. C. (2018). N eural and behavioral correlates of negative self-focused thought associated with depression. *Human Brain Mapping,**39*(5), 2246–2257. 10.1002/hbm.24003 29427365 10.1002/hbm.24003 PMC5895491

[CR48] Pizzagalli, D. A., & Roberts, A. C. (2022). Prefrontal cortex and depression. *Neuropsychopharmacology,**47*(1), 225–246. 34341498 10.1038/s41386-021-01101-7 PMC8617037

[CR49] Pulopulos, M. M., De Witte, S., Vanderhasselt, M.-A., De Raedt, R., Schiettecatte, J., Anckaert, E., Salvador, A., & Baeken, C. (2019). The influence of personality on the effect of iTBS after being stressed on cortisol secretion. *PLoS One,**14*(10), e0223927. 31618272 10.1371/journal.pone.0223927 PMC6795454

[CR50] Pulopulos, M. M., Schmausser, M., De Smet, S., Vanderhasselt, M.-A., Baliyan, S., Venero, C., Baeken, C., & De Raedt, R. (2020). The effect of HF-rTMS over the left DLPFC on stress regulation as measured by cortisol and heart rate variability. *Hormones and Behavior,**124*, 104803. 32526225 10.1016/j.yhbeh.2020.104803

[CR51] R Core Team. (2023). *R: A language and environment for statistical computing*. R foundation for statistical computing. https://www.R-project.org/

[CR52] Ray, R. D., Ochsner, K. N., Cooper, J. C., Robertson, E. R., Gabrieli, J. D., & Gross, J. J. (2005). Individual differences in trait rumination and the neural systems supporting cognitive reappraisal. *Cognitive, Affective, & Behavioral Neuroscience,**5*, 156–168. 10.3758/cabn.5.2.156 16180622

[CR53] Remue, J., Vanderhasselt, M.-A., Baeken, C., Rossi, V., Tullo, J., & De Raedt, R. (2016). The effect of a single HF-rTMS session over the left DLPFC on the physiological stress response as measured by heart rate variability. *Neuropsychology,**30*(6), 756. 26618798 10.1037/neu0000255

[CR54] Rosenbaum, D., Hilsendegen, P., Thomas, M., Haeussinger, F. B., Nuerk, H.-C., Fallgatter, A. J., Nieratschker, V., Ehlis, A.-C., & Metzger, F. G. (2018a). Disrupted prefrontal functional connectivity during post-stress adaption in high ruminators. *Scientific Reports,**8*(1), 15588. 10.1038/s41598-018-33777-1 30348981 10.1038/s41598-018-33777-1 PMC6197217

[CR55] Rosenbaum, D., Thomas, M., Hilsendegen, P., Metzger, F. G., Haeussinger, F. B., Nuerk, H.-C., Fallgatter, A. J., Nieratschker, V., & Ehlis, A.-C. (2018b). Stress-related dysfunction of the right inferior frontal cortex in high ruminators: An fNIRS study. *NeuroImage: Clinical,**18*, 510–517. 10.1016/j.nicl.2018.02.022 29560307 10.1016/j.nicl.2018.02.022 PMC5857918

[CR56] Rosenbaum, D., Int-Veen, I., Laicher, H., Torka, F., Kroczek, A., Rubel, J., Lawyer, G., Bürger, Z., Bihlmaier, I., Storchak, H., et al. (2021). Insights from a laboratory and naturalistic investigation on stress, rumination and frontal brain functioning in MDD: An fNIRS study. *Neurobiology of Stress,**15*, 100344. 34124320 10.1016/j.ynstr.2021.100344 PMC8173308

[CR57] Rothwell, J. C., Hallett, M., Berardelli, A., Eisen, A., Rossini, P., & Paulus, W. (1999). Magnetic stimulation: Motor evoked potentials. the international federation of clinical neurophysiology. *Electroencephalography and Clinical Neurophysiology Supplement,**52*, 97–103. 10590980

[CR58] Sassaroli, A., & Fantini, S. (2004). Comment on the modified Beer-Lambert law for scattering media. *Physics in Medicine & Biology,**49*(14), N255. 15357206 10.1088/0031-9155/49/14/n07

[CR59] Sathappan, A. V., Luber, B. M., & Lisanby, S. H. (2019). The dynamic duo: Combining noninvasive brain stimulation with cognitive interventions. *Progress in Neuro-Psychopharmacology and Biological Psychiatry,**89*, 347–360. 30312634 10.1016/j.pnpbp.2018.10.006

[CR60] Schommer, N. C., Hellhammer, D. H., & Kirschbaum, C. (2003). Dissociation between reactivity of the hypothalamus-pituitary-adrenal axis and the sympathetic-adrenal-medullary system to repeated psychosocial stress. *Psychosomatic Medicine,**65*(3), 450–460. 10.1097/01.PSY.0000035721.12441.17 12764219 10.1097/01.psy.0000035721.12441.17

[CR61] Schutter, D. (2009). Antidepressant efficacy of high-frequency transcranial magnetic stimulation over the left dorsolateral prefrontal cortex in double-blind sham-controlled designs: A meta-analysis. *Psychological Medicine,**39*(1), 65–75. 18447962 10.1017/S0033291708003462

[CR62] Siegle, G. J., Steinhauer, S. R., Thase, M. E., Stenger, V. A., & Carter, C. S. (2002). Can’t shake that feeling: Event-related fMRI assessment of sustained amygdala activity in response to emotional information in depressed individuals. *Biological Psychiatry,**51*(9), 693–707. 11983183 10.1016/s0006-3223(02)01314-8

[CR63] Singh, A. K., Okamoto, M., Dan, H., Jurcak, V., & Dan, I. (2005). Spatial registration of multichannel multi-subject fNIRS data to MNI space without MRI. *Neuroimage,**27*(4), 842–851. 15979346 10.1016/j.neuroimage.2005.05.019

[CR64] Smith, J. E., & Peterchev, A. V. (2018). Electric field measurement of two commercial active/sham coils for transcranial magnetic stimulation. *Journal of Neural Engineering,**15*(5), 054001. 29932429 10.1088/1741-2552/aace89 PMC6125221

[CR65] Studio Team. (2022). *RStudio: Integrated development for R*. RStudio, Inc. https://www.rstudio.com/

[CR66] Treynor, W., & Gonzalez, R. (2003). Rumination Reconsidered: A Psychometric Analysis. *Cognitive Therapy and Research*, *27*. 10.1023/A:1023910315561

[CR67] Watkins, E. R., & Roberts, H. (2020). Reflecting on rumination: Consequences, causes, mechanisms and treatment of rumination. *Behaviour Research and Therapy,**127*, 103573. 10.1016/j.brat.2020.103573 32087393 10.1016/j.brat.2020.103573

[CR68] Watson, D., Clark, L. A., & Tellegen, A. (1988). Development and validation of brief measures of positive and negative affect: The PANAS scales. *Journal of Personality and Social Psychology,**54*(6), 1063–1070. 10.1037/0022-3514.54.6.1063 3397865 10.1037//0022-3514.54.6.1063

[CR69] Wickham, H. (2016). *ggplot2: Elegant graphics for data analysis*. Springer-Verlag. https://ggplot2.tidyverse.org

