# Supplementary material for: Investigating the neural and behavioral correlates of the stress-rumination link in healthy humans by modulating the left Dorsolateral Prefrontal Cortex using Theta Burst Stimulation
Source: Cogn Affect Behav Neurosci. 2025 Jun 23;25(5):1449–72. doi: 10.3758/s13415-025-01305-0 (PMC12464127; doi:10.3758/s13415-025-01305-0)
Supplement: Supplementary file 1 — Supplementary file1 (PDF 2.72 MB) [file 13415_2025_1305_MOESM1_ESM.pdf]

Supplementary material to:

Investigating the neural and behavioral correlates of the stress-rumination link in healthy humans by modulating the left Dorsolateral Prefrontal Cortex using Theta Burst Stimulation

Isabell Int-Veen[1], Ute Eßer[1,2], Sandra Ladegast[1,2], Leonhard Liermann[1], Ramona Täglich[1], Betti Schopp[1], Hans-Christoph Nuerk[2,3], Christian Plewnia[1,3], Agnes Kroczek[1], Stefanie De Smet[4,5,6], Marie-Anne Vanderhasselt[4,5], Andreas J. Fallgatter[1,3,7], Ann-Christine Ehlis[1,3,7], Beatrix Barth[1,3,7*], David Rosenbaum[1*]

[1] Tübingen Center for Mental Health (TüCMH), Department of Psychiatry and Psychotherapy, University Hospital Tübingen, Tübingen, Germany
[2] Department of Psychology, University of Tübingen, Germany
[3] German Center for Mental Health (DZPG), partner site Tübingen, Germany
[4] Department of Head and Skin, Psychiatry and Medical Psychology, Ghent University Hospital, Ghent University, Ghent, Belgium
[5] Ghent Experimental Psychiatry (GHEP) lab, Ghent, Belgium
[6] Brain Stimulation and Cognition (BSC) Lab, Department of Cognitive Neuroscience, Faculty of Psychology & Neuroscience, Maastricht University, Maastricht - The Netherlands
[7] LEAD Graduate School & Research Network, University of Tübingen, Tübingen, Germany

Corresponding Author:
Isabell Int-Veen
Calwerstraße 14
72076 Tübingen
Germany

email: isabell.int-veen@med.uni-tuebingen.de

Supplementary material S1: Inclusion and exclusion criteria

Inclusion criteria:

- age between 18 and 50 years
- normal vision (or appropriate correction)
- right-handedness
- no metal in the skull or brain
- German as native language or very good knowledge of German

Exclusion criteria:

- diabetes mellitus
- renal insufficiency
- untreated hypertension
- history of traumatic brain injury
- cardiac arrhythmia
- acute substance abuse
- adrenal insufficiency
- any acute psychiatric or neurological disorder (including any anomalies in the SCID-Screening (Structured Clinical Interview; First et al., 2015)
- in case of women: pregnancy

Supplementary material S2: Information on the a priori power analysis

Currently, only one study exists that investigated the effects of TBS on state rumination increases through the TSST. In the study of De Witte et al. (2020), *N* = 40 participants were either stimulated with iTBS or sham TBS after the TSST was applied. Note that the study also used a within-subject design in which all participants received iTBS and sham after the TSST was conducted on two separate days. In their study, the authors found an increase of state rumination through the TSST and a marginally significant interaction between trait rumination and the TBS protocol on increases of state rumination (De Witte et al., 2020). The statistical parameters are given as *B* = -.32, *t*(36) = - 1.891, *p* = .067. Thankfully, the authors sent us the data of this study and we therefore were able to compute the effect size of this effect directly from the data, which was η_p^2 = .107 (approx. *d* = .69, *f* =.34). However, it is important to note that the study didn't use a stratified sample of trait rumination or clinical participants. As a result, only 8 of 38 subjects fulfilled our previous criteria for high ruminators (Rosenbaum, Hilsendegen, et al., 2018; Rosenbaum, Thomas, et al., 2018). As we showed in our previous studies (Rosenbaum et al., 2021, 2024; Rosenbaum, Hilsendegen, et al., 2018; Rosenbaum, Thomas, et al., 2018) that state rumination increased in high trait ruminators/patients more than in low trait ruminators/healthy controls, we would assume that in stratified and clinical samples the above-noted buffering effect of the TBS protocol could be even higher, as the general reactivity of these samples in state rumination (under sham stimulation) is stronger than in the study of De Witte et al. (2020).

However, as seen in the study of De Witte et al. (2020), it is important to achieve high power as the effect was only marginally significant. Therefore, we aim to assess *N* = 80 subjects which allows to detect between-within subject interactions up to *f* = .15 for the effect of state rumination, as computed with G*Power 3.1.9.2 (with a power of 1-β = .95, α = .05. *r* = .7; between measurements, assessed on the basis of data from our previous studies). To compensate for data loss and drop-outs, we plan to collect an additional 10% (*n* = 8) of participants to the computed sample size, resulting in *N* = 88 participants.

Supplementary material S3: Consort diagram

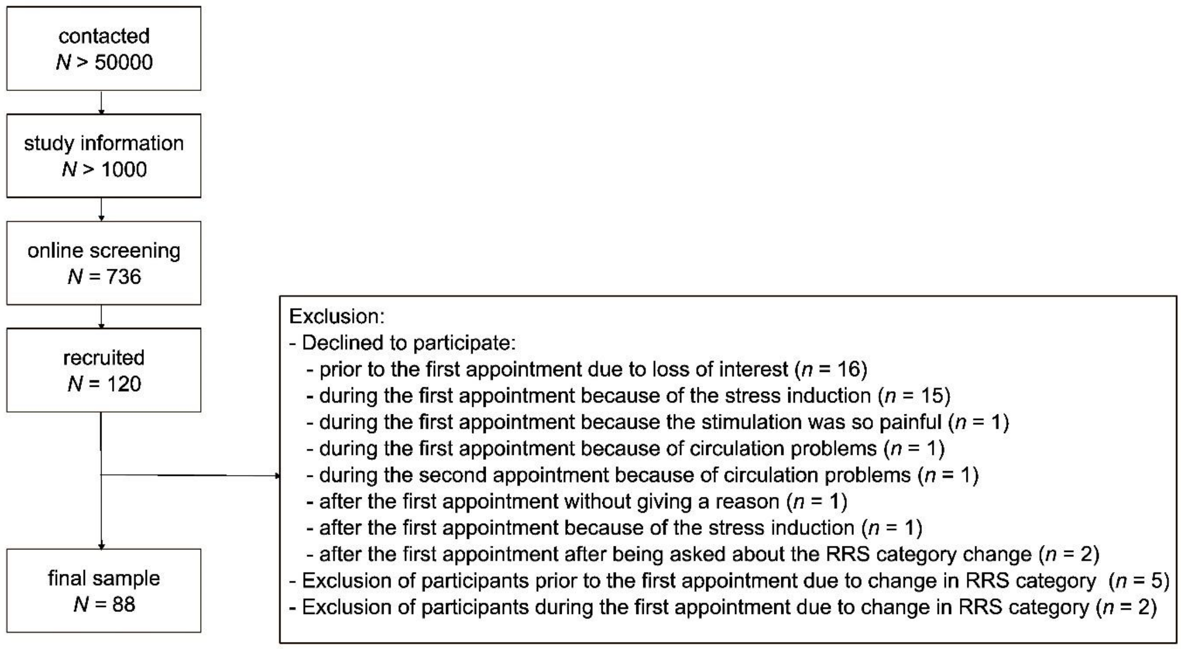

Supplementary material S4: Items of the state rumination questionnaire

Items of the state rumination questionnaire including adapted items from the Ruminative Response Scale (Nolen-Hoeksema & Morrow, 1991), Amsterdam Resting-State Questionnaire (Diaz et al., 2013) and the Perseverative Thinking Questionnaire (Ehring et al., 2011). Subjects were instructed to rate if the items were in line with their mental state during the last resting-state measurement.

item	German	English
1	Ich dachte immer wieder an meine Probleme.	I repeatedly thought about my problems.
2	Ich verharrte im Denken an Dinge, die mich beunruhigen.	I kept thinking about things that bother me.
3	Meine Gedanken wiederholten sich, ohne dass ich zu einer Lösung kam.	I dwelled on my thoughts without coming to a solution.
4	Ich verlor mich in meinen negativen Gedanken.	I got lost in my negative thoughts.
5	Ich konnte meine Gedanken nur mühsam festhalten.	I had difficulties holding on to my thoughts.
6	Ich konnte mich nicht von meinen negativen Gedanken lösen.	I could not let go of my negative thoughts.
7	Ich war bei der Sache.	I was present.
8	Ich dachte darüber nach, warum ich mich in bestimmten Situationen falsch verhalten habe.	I thought about why I acted wrong in certain situations.
9	Ich fragte mich, warum ich Probleme habe, die andere nicht haben.	I thought why I have problems other people don't have.
10	Ich fragte mich, womit ich meine momentane Lebenssituation verdient habe.	I thought about whereby I deserved my current life situation.
11	Ich dachte darüber nach, warum ich die Dinge nicht besser in den Griff bekomme.	I thought why I can't handle things better.
12	Ich dachte an all meine Defizite und Misserfolge, Macken und Fehler.	I thought about all my shortcomings, failings, faults, mistakes.
13	Ich konnte flexibel zwischen meinen Gedanken hin und her schalten.	I could switch between my thoughts flexibly.
14	Ich dachte an vergangene Situationen, die ich bereue.	I thought about past situations that I regret.
15	Ich machte mir Selbstvorwürfe.	I blamed myself.
16	Ich verlor mich in Gedanken an Vergangenes.	I got lost in thoughts about the past.
17	Ich war von meinen Problemen und Sorgen stark vereinnahmt.	I was consumed by my problems and worries.
18	Meine negativen Gedanken ließen mich nicht los.	I couldn't let go of my negative thoughts.

Supplementary material S5: Illustration of the approximation of the TBS-induced electric field

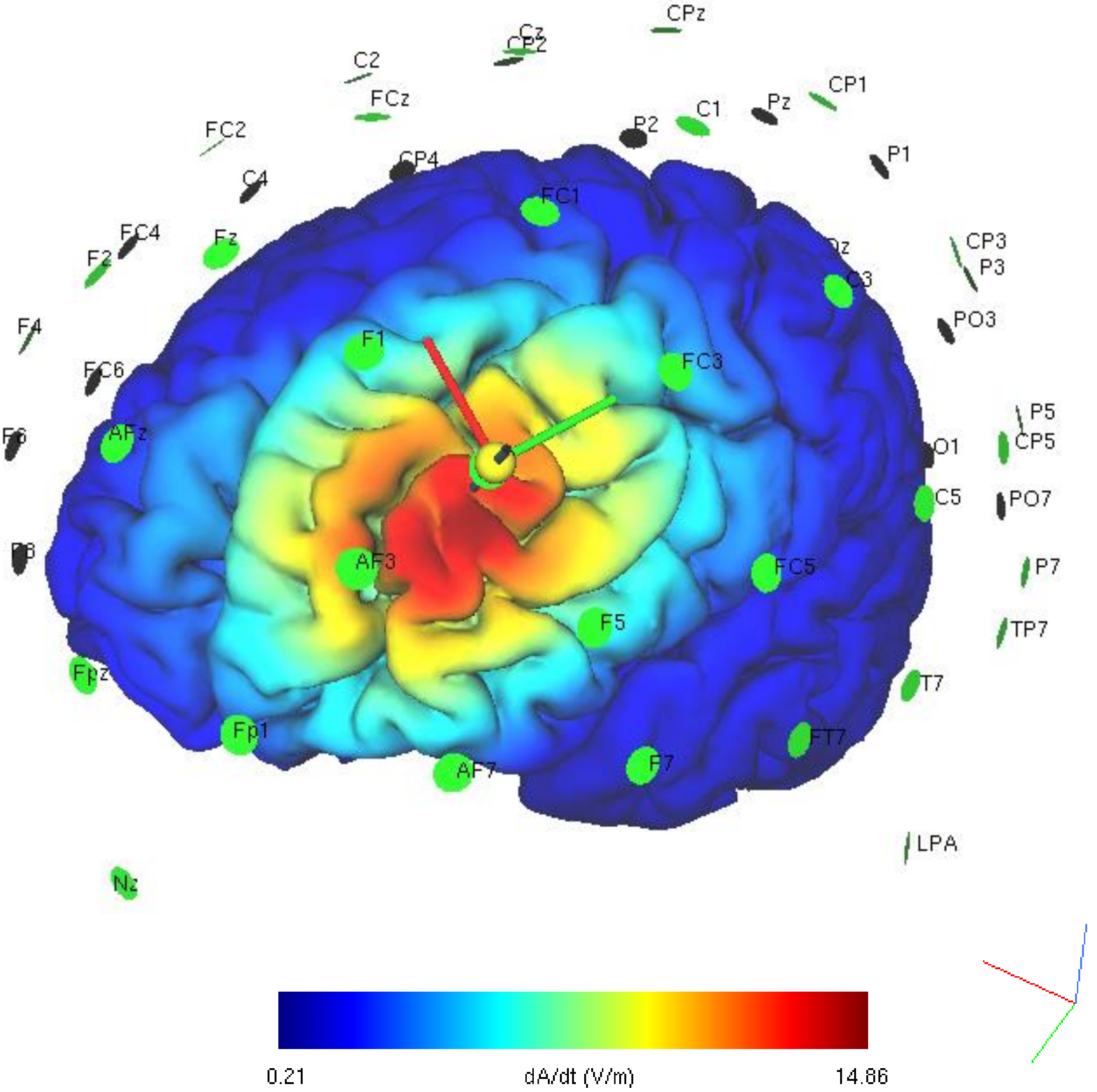

Figure S5. Approximation of the TBS-induced electric field. The coil (MagVenture Cool-B65 Active/Placebo coil) was positioned over the left DLPFC, corresponding to the F3 electrode according to the 10–20 EEG system. The color red indicates the strongest electric field. The y-axis in green indicates the direction of the coil handle. This plot was created using SimNIBS 4.1.0. (Thielscher et al., 2015)

Supplementary material S6: Placement of the three probesets.

Please note that the corresponding numbers represent the channels (located midway between emitter and detector).

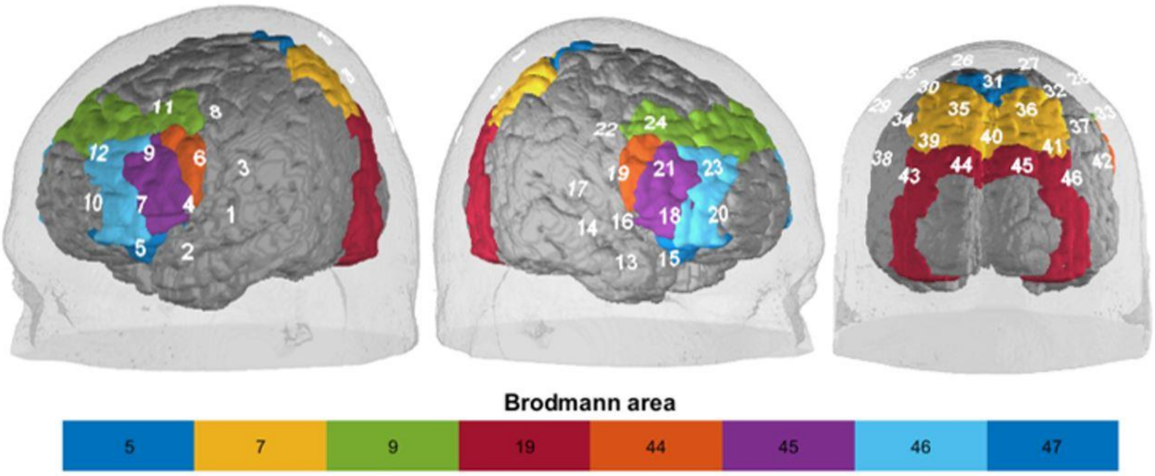

Supplementary material S7: Definition of Regions of Interest

ROI	channel
left VLPFC	7 9 6
right VLPFC	18 21 19
left DLPFC	10 12 11
right DLPFC	20 23 24
SAC	27 26 25 28 30 31 32 35 36

Note. VLPFC = Ventrolateral Prefrontal Cortex, DLPFC = Dorsolateral Prefrontal Cortex, SAC = Somatosensory Association Cortex. Please note that the TBS was applied over channel 12 which corresponds to F3.

Supplementary material S8: Results of positive affect, math performance and heart rate

Positive affect contrasts

According to Mahalanobis distances (p < .001), there were no multivariate outliers for positive affect ratings but 9 participants with missing data. Fitting the rmANOVA, we observed a significant interaction of time and order of stimulation conditions, $F(3, 213) = 10.566$, $p < .001$, $\eta_p^2 = .130$, and a marginally significant interaction of time and group, $F(3, 213) = 2.156$, $p = .094$, $\eta_p^2 = .029$.

We then fitted separate rmANOVAs dependent on the order of stimulation conditions. As a result, we observed only a significant main effect of time (sham → active: $F(2.577, 92.767) = 5.887$, $p < .01$, $\eta_p^2 = .141$, active → sham: $F(3, 105) = 4.764$, $p < .01$, $\eta_p^2 = .120$).

Polynomial contrasts revealed a linear relationship in both cases but, again, in different directions depending on the order of stimulation conditions (active → sham: $F(1, 35) = 11.211$, $p < .01$, $\eta_p^2 = .243$; sham → active: $F(1, 36) = 10.481$, $p < .01$, $\eta_p^2 = .225$). For participants having received sham stimulation first, the differences between active and sham stimulation increased over the course of the experiment while they decreased in case participants received active stimulation first (see figure S7.1).

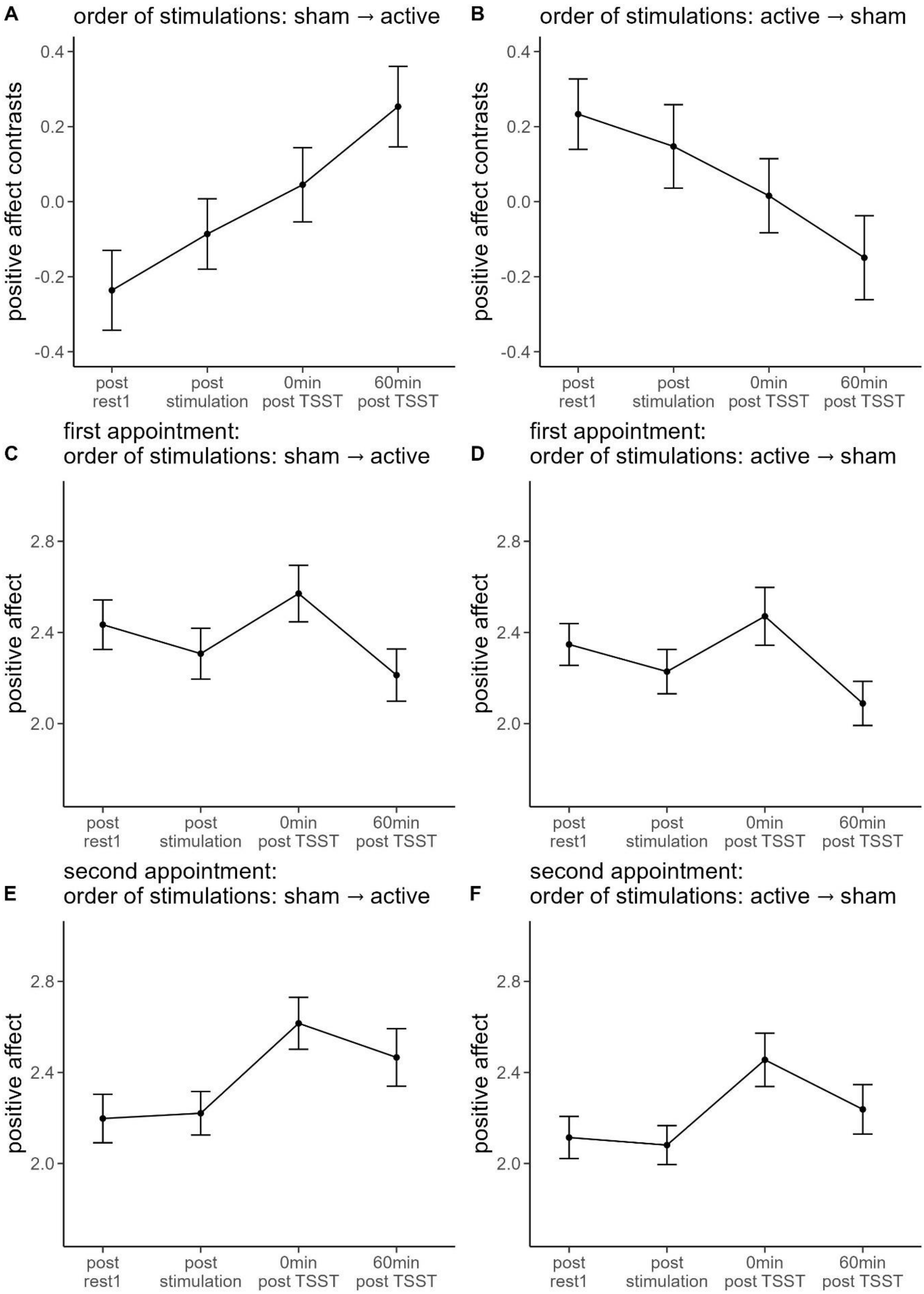

Figure S7.1. Line plot of the contrasts of positive affect ratings dependent on order of conditions (A = sham stimulation at the first appointment, active stimulation at the second appointment; B = active stimulation at the first appointment, sham stimulation at the second appointment) and line plot of raw data of positive affect ratings dependent on order of conditions and appointment (C = first appointment sham stimulation; D = first appointment active stimulation; E = second appointment active stimulation; F = second appointment sham stimulation). rest = resting-state measurement, sTBS = sham theta burst stimulation, cTBS = continuous theta burst stimulation, iTBS = intermittent theta burst stimulation, 0min post TSST = 0min after the Trier Social Stress Test. Error bars indicate 1 standard error of the mean.

Mean number of performed calculations contrasts

According to Mahalanobis distances (p < .001), there were no multivariate outliers for the number of performed calculations. Fitting the rmANOVA, we observed a significant interaction effect of time and the order of stimulation conditions, F(1.817, 145.388) = 61.208, p < .001, η_p^2 = .433, as well as a significant lower-order main effect of the order of stimulation conditions, F(1, 80) = 118.729, p < .001, η_p^2 = .597. We further found a marginally significant interaction of group and order of stimulation conditions, F(1, 80) = 3.900, p = .052, η_p^2 = .046, and a marginally significant main effect of stimulation condition, F(1, 80) = 3.023, p = .086, η_p^2 = .036.

We then fitted separate rmANOVAs dependent on the order of stimulation conditions. As a result, we observed a significant main effect of time (sham → active: F(1.764, 70.573) = 30.964, p < .001, η_p^2 = .436; active → sham: F(1.895, 75.811) = 30.990, p < .001, η_p^2 = .437) as well as a significant intercept (sham → active: F(1, 40) = 77.755, p < .001, η_p^2 = .660; active → sham: F(1, 40) = 48.773, p < .001, η_p^2 = .549) as well as a marginally significant main effect of stimulation condition but only in case participants received active stimulation first, F(1, 40) = 3.133, p = .084, η_p^2 = .073.

Polynomial contrasts of the main effect of time indicated a quadratic time course of the contrasts but in opposing directions depending on the order of stimulation conditions (active → sham: F(1, 40) = 21.297, p < .001, η_p^2 = .347; sham → active: F(1, 40) = 46.159, p < .001, η_p^2 = .536). Initially, the mean number of read-out numbers (control task 1) was comparable between active and sham stimulation, regardless of the order of stimulation conditions. For participants having received sham stimulation first, we observed positive contrasts during control task 2 and the arithmetic task of the TSST. That means, participants performed more calculations following the active stimulation compared to the sham stimulation. The opposite was true for participants having received active stimulation first (see figure S7.2).

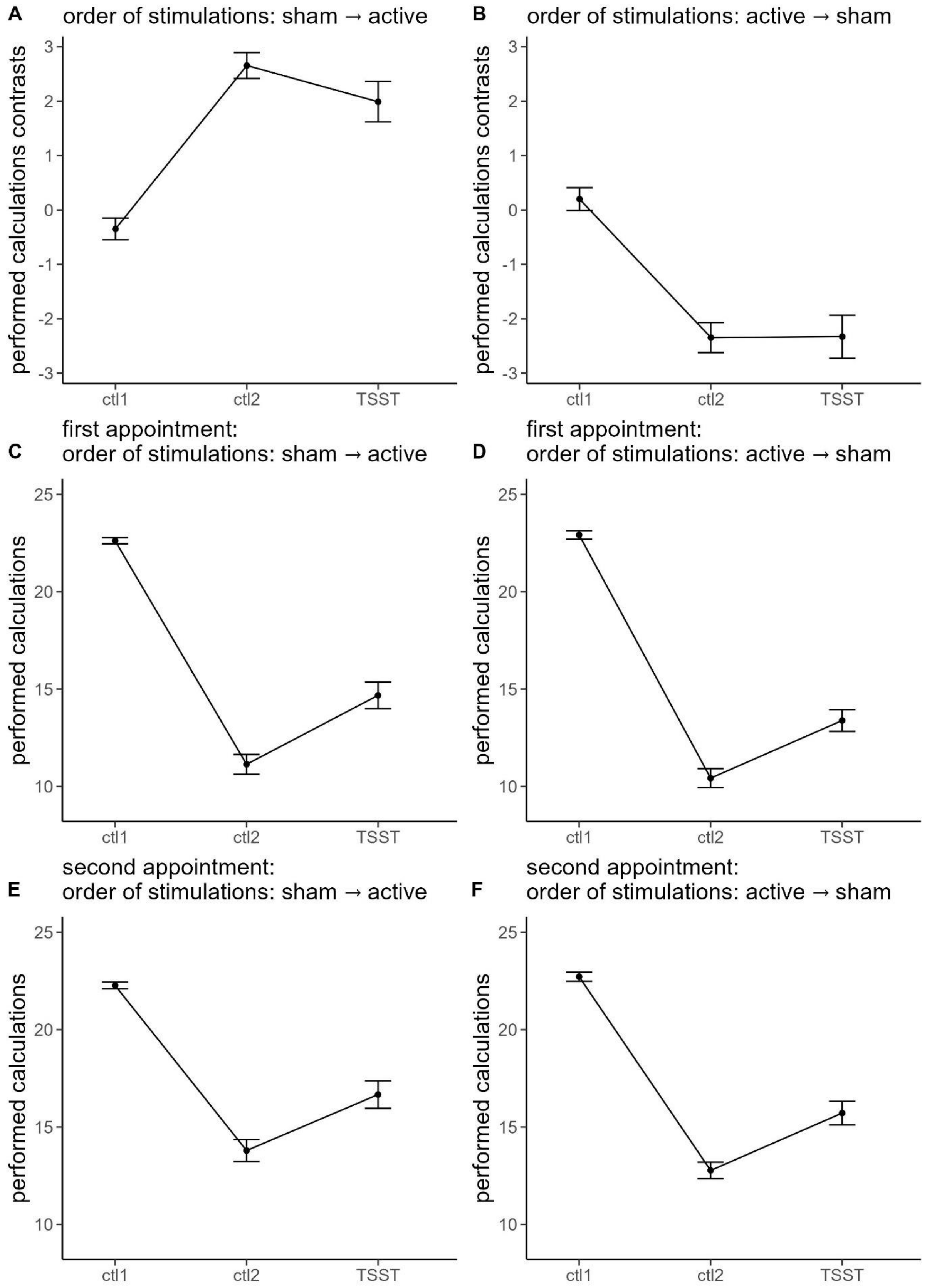

Figure S7.2. Line plot of the contrasts of the number of performed calculations dependent on order of conditions (A = sham stimulation at the first appointment, active stimulation at the second appointment; B = active stimulation at the first appointment, sham stimulation at the second appointment) and line plot of raw data of performed calculations dependent on order of conditions and appointment (C = first appointment sham stimulation; D = first appointment active stimulation; E = second appointment active stimulation; F = second appointment sham stimulation). RRS = Ruminative Response Scale, sTBS = sham theta burst stimulation, cTBS = continuous theta burst stimulation, iTBS = intermittent theta burst stimulation, ctl1 = control task1, ctl2 = control task 2, TSST = arithmetic task of the TSST; Error bars indicate 1 standard error of the mean.

Total number of errors contrasts

Checking for multivariate outliers using Mahalanobis distances (p < .001), we excluded the data of 2 participants. Fitting the rmANOVA, we observed a significant three-way interaction of time, group and order of stimulation conditions, F(1.646, 128.426) = 4.174, p < .05, η_p^2 = .051, a significant lower-order interaction of time and the order of stimulation conditions, F(1.646, 128.426) = 11.746, p < .001, η_p^2 = .131, as well as a significant main effect of the order of stimulation conditions, F(1, 78) = 11.670, p < .01, η_p^2 = .130.

We then fitted the same rmANOVA dependent on order of stimulation conditions. As a result, we observed a significant interaction of time and group in case participants received active stimulation first, F(1.606, 61.038) = 4.116, p < .05, η_p^2 = .098 (see figure S7.3B), as well as a marginally significant interaction of time and stimulation condition, F(1.606, 61.038) = 2.702, p = .086, η_p^2 = .066. For participants having received sham stimulation first, we only observed a significant main effect of time, F(1.710, 68.391) = 7.080, p < .01, η_p^2 = .150 and significant main effect of the constant term, F(1, 40) = 11.273, p < .01, η_p^2 = .220.

Polynomial contrast of the interaction of time and group indicated linear association, F(1, 38) = 6.692, p < .05, η_p^2 = .150: While for both groups, low and high ruminators, the number of errors was comparable between active and sham stimulation during control task 1 and 2, low ruminators made more errors following active stimulation (AP1) and less errors following sham stimulation (AP2) during the arithmetic task of the TSST while the number of errors stayed the same for high ruminators.

Benjamini-Hochberg-corrected post-hoc tests indicated no significant differences between the groups at any given time point.

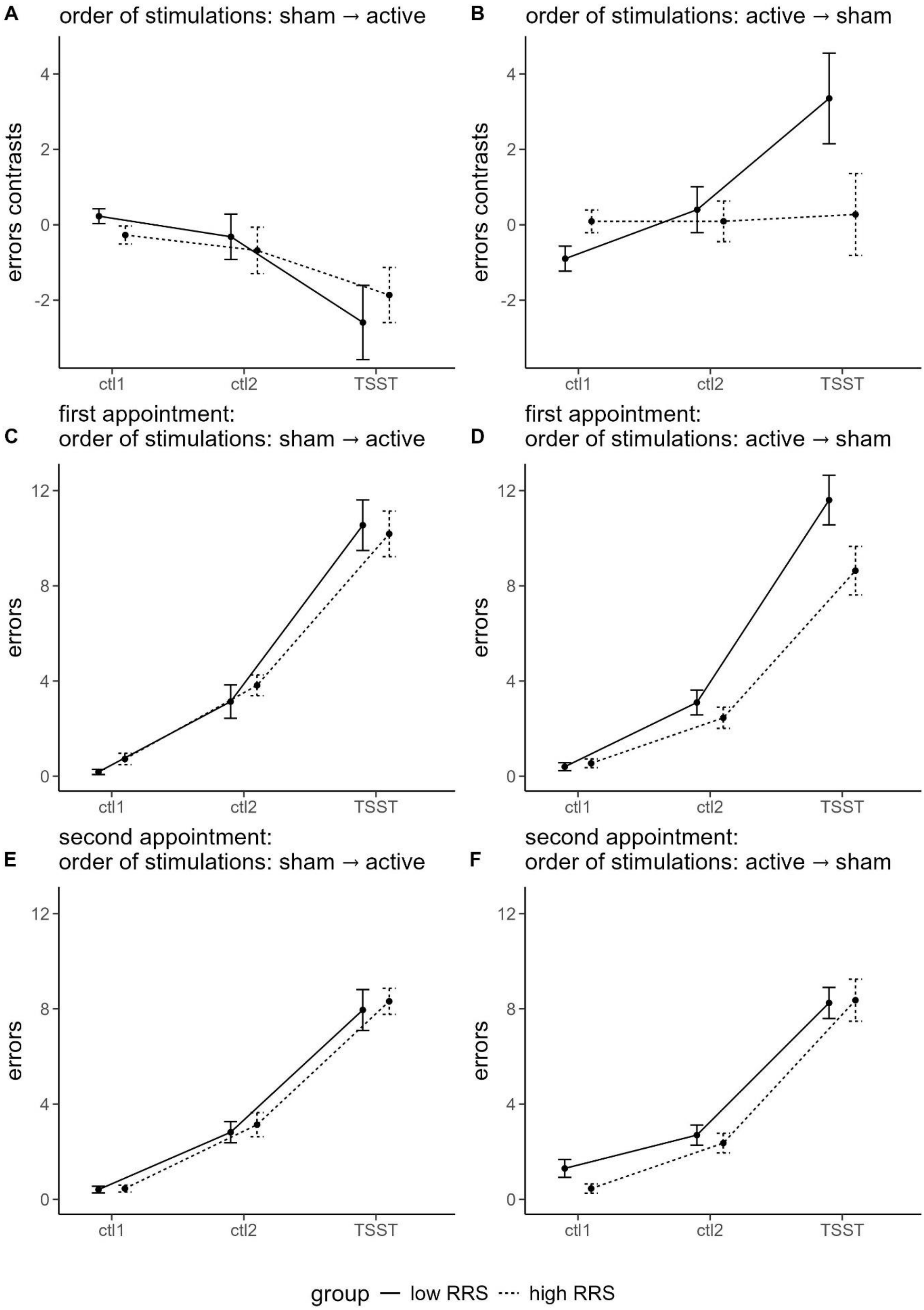

Figure S7.3. Line plot of the contrasts of the number of errors made dependent on order of conditions (A = sham stimulation at the first appointment, active stimulation at the second appointment; B = active stimulation at the first appointment, sham stimulation at the second appointment) and line plot of raw data of the number of errors made dependent on order of conditions and appointment (C = first appointment sham stimulation; D = first appointment active stimulation; E = second appointment active stimulation; F = second appointment sham stimulation). RRS = Ruminative Response Scale, sTBS = sham theta burst stimulation, cTBS = continuous theta burst stimulation, iTBS = intermittent theta burst stimulation, ctl1 = control task1, ctl2 = control task 2, TSST = arithmetic task of the TSST; Error bars indicate 1 standard error of the mean.

Heart rate contrasts

Checking for multivariate outliers using Mahalanobis distances ($p < .001$), we excluded the data of 4 participants and 7 missing data. Fitting a rmANOVA, we observed a significant interaction of time and order of stimulation conditions, $F(3.558, 245.519) = 4.959$, $p < .01$, $\eta_p^2 = .067$, as well as a marginally significant main effect of order of stimulation conditions, $F(1, 69) = 2.855$, $p = .096$, $\eta_p^2 = .040$.

Fitting the same rmANOVA separately for participants dependent on order of stimulation conditions yielded a significant main effect of time (sham → active: $F(3.075, 101.459) = 3.204$, $p < .05$, $\eta_p^2 = .088$; active → sham: $F(3.620, 139.331) = 2.611$, $p < .05$, $\eta_p^2 = .068$), as well as a marginally significant main effect of the intercept but only in case participants received sham first, $F(1, 33) = 3.925$, $p = .056$, $\eta_p^2 = .106$.

Investigating the main effect of time using polynomial contrasts, yielded a linear time course in both cases (sham → active: $F(1, 40) = 11.446$, $p < .01$, $\eta_p^2 = .222$; active → sham: $F(1, 38) = 7.942$, $p < .01$, $\eta_p^2 = .173$) (see figure S7.4).

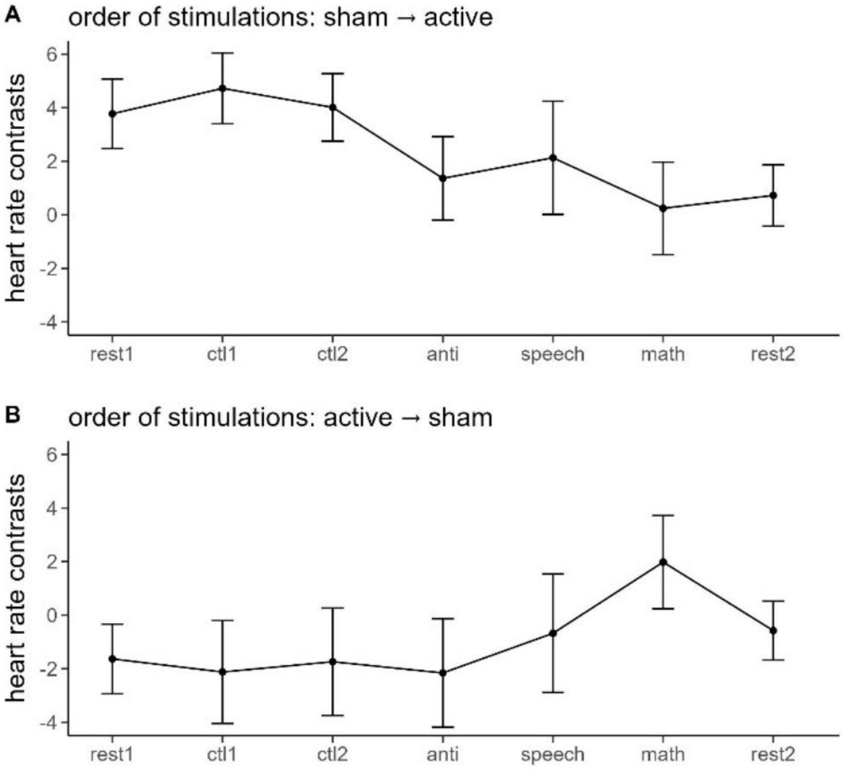

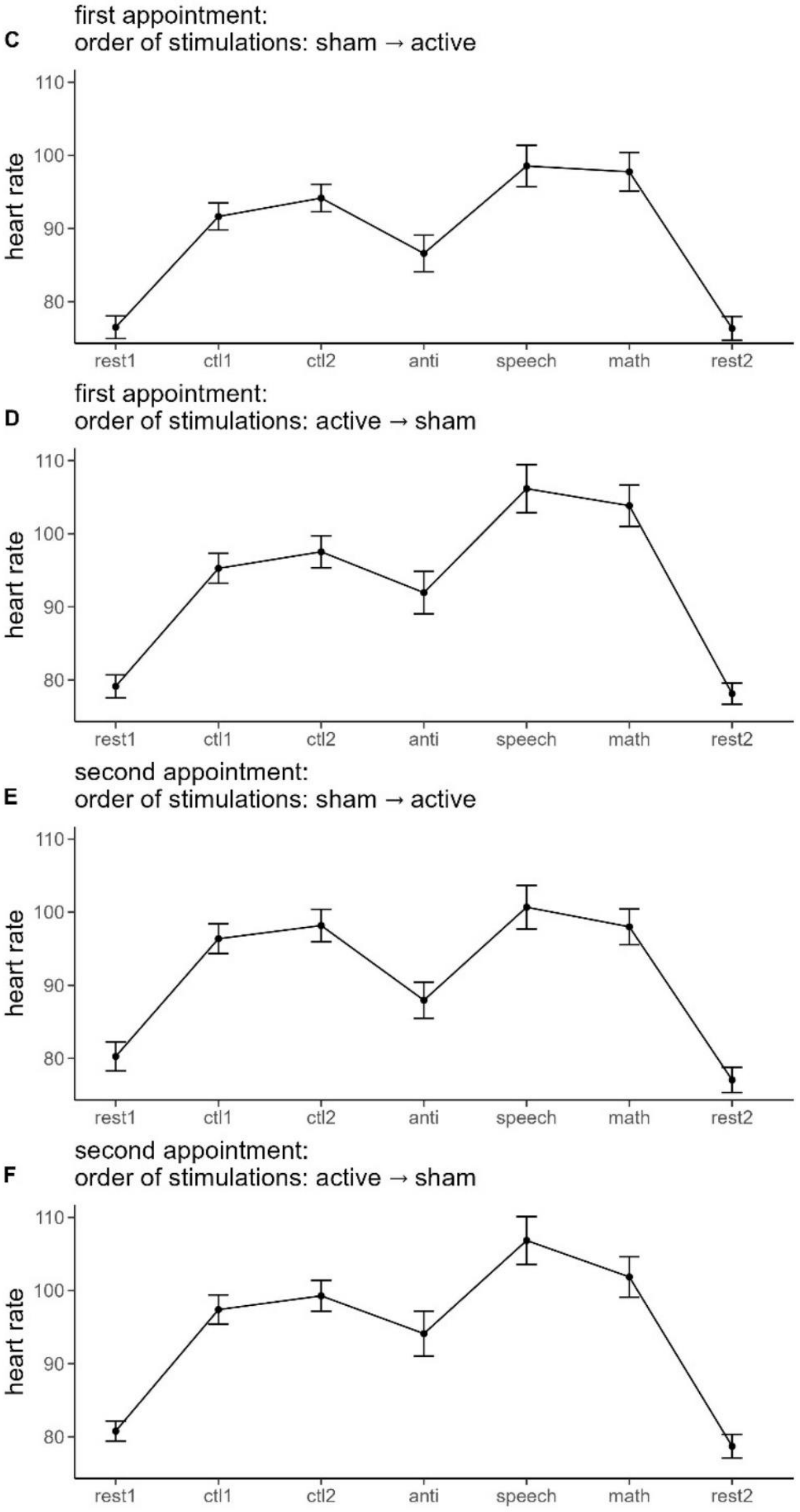

Figure S7.4. Line plot of the contrasts of mean heart rates in beats per minute dependent on order of conditions (A = sham stimulation at the first appointment, active stimulation at the second appointment; B = active stimulation at the first appointment, sham stimulation at the second appointment) and line plot of raw data of mean heart rates in beats per minute dependent on order of conditions and appointment (C = first appointment sham stimulation; D = first appointment active stimulation; E = second appointment active stimulation; F = second appointment sham stimulation). RRS = Ruminative Response Scale, sTBS = sham theta burst stimulation, cTBS = continuous theta burst stimulation, iTBS = intermittent theta burst stimulation, rest1 = resting-state measurement 1, ctl1 = control task1, ctl2 = control task 2, anti = anticipation phase of the TSST, speech = job interview of the TSST, math = arithmetic task of the TSST; Error bars indicate 1 standard error of the mean.

Supplementary material S9: Illustration of Reliable Change Indices

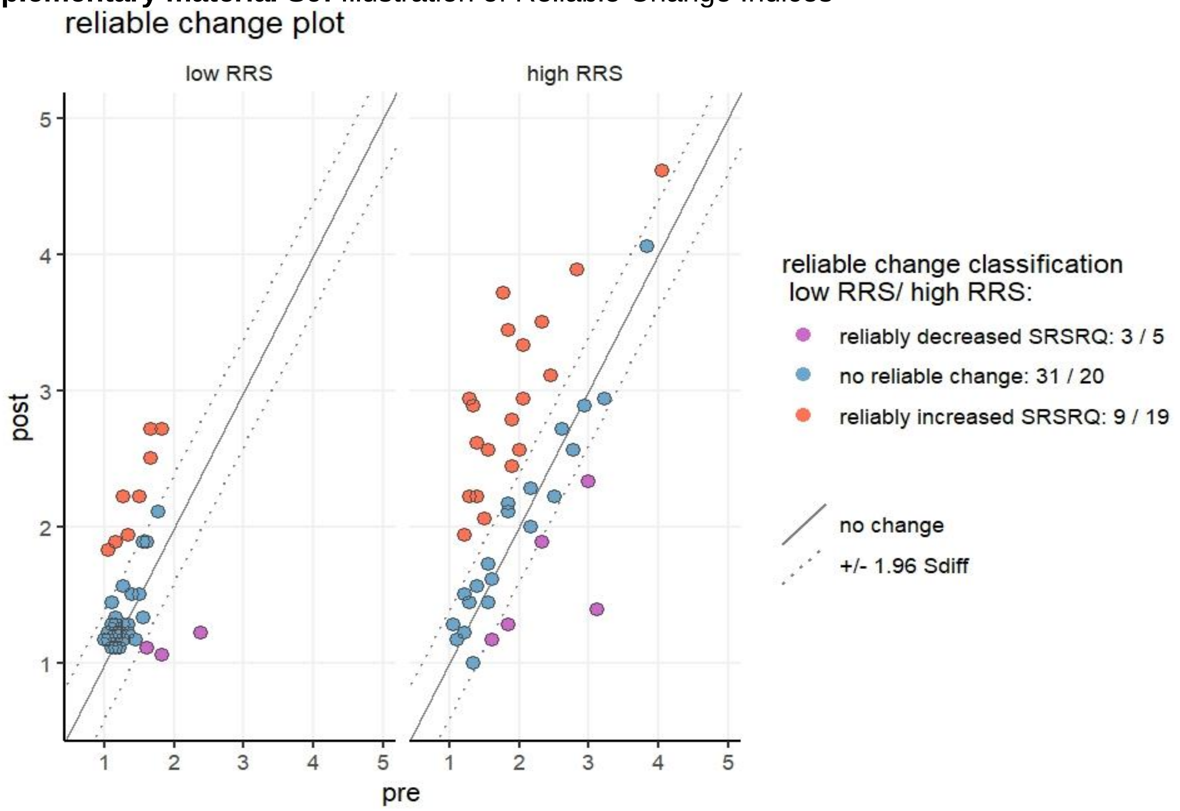

Figure S9.1 Reliable change index plotted for changes in state rumination previous to the stress induction (rest1) to after the stress induction (rest2) for the first appointment dependent on RRS group.

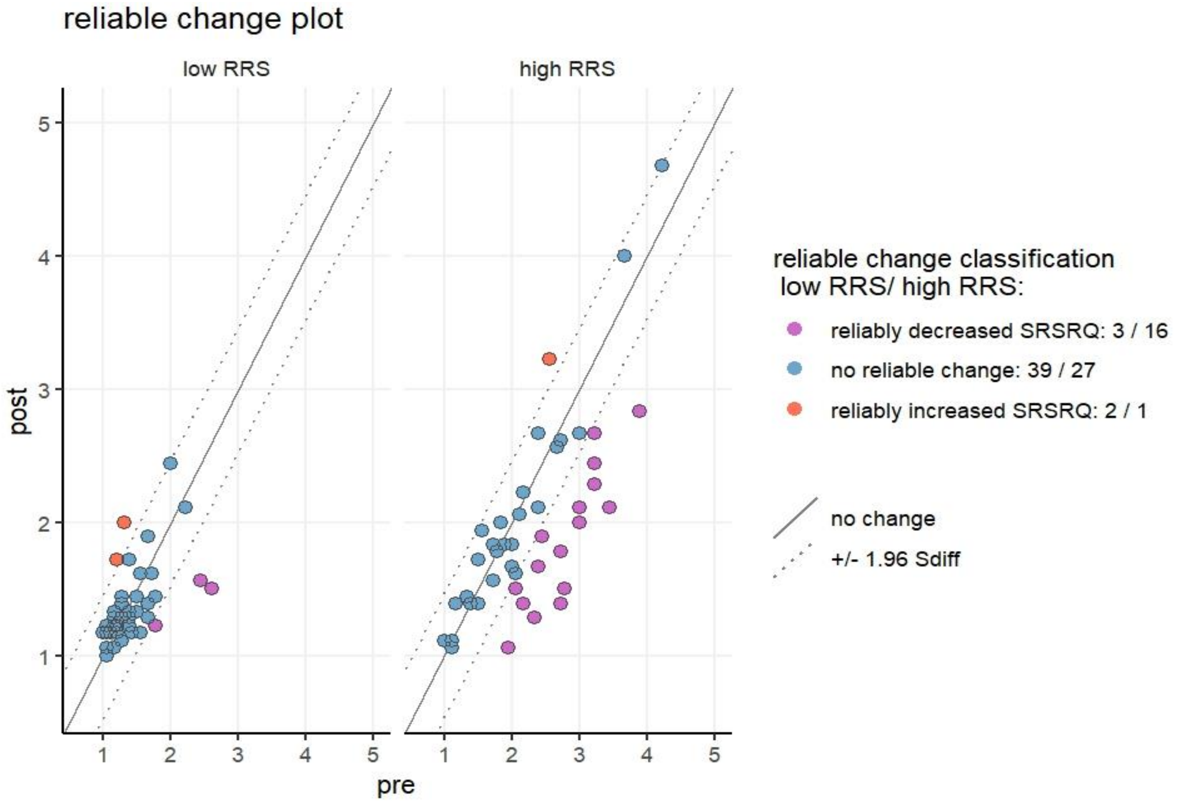

Figure S9.2 Reliable change index plotted for changes in state rumination previous to the stress induction (rest1) to after the stress induction (rest2) for the second appointment dependent on RRS group.

Supplementary material S10: Illustraction of Brainmaps

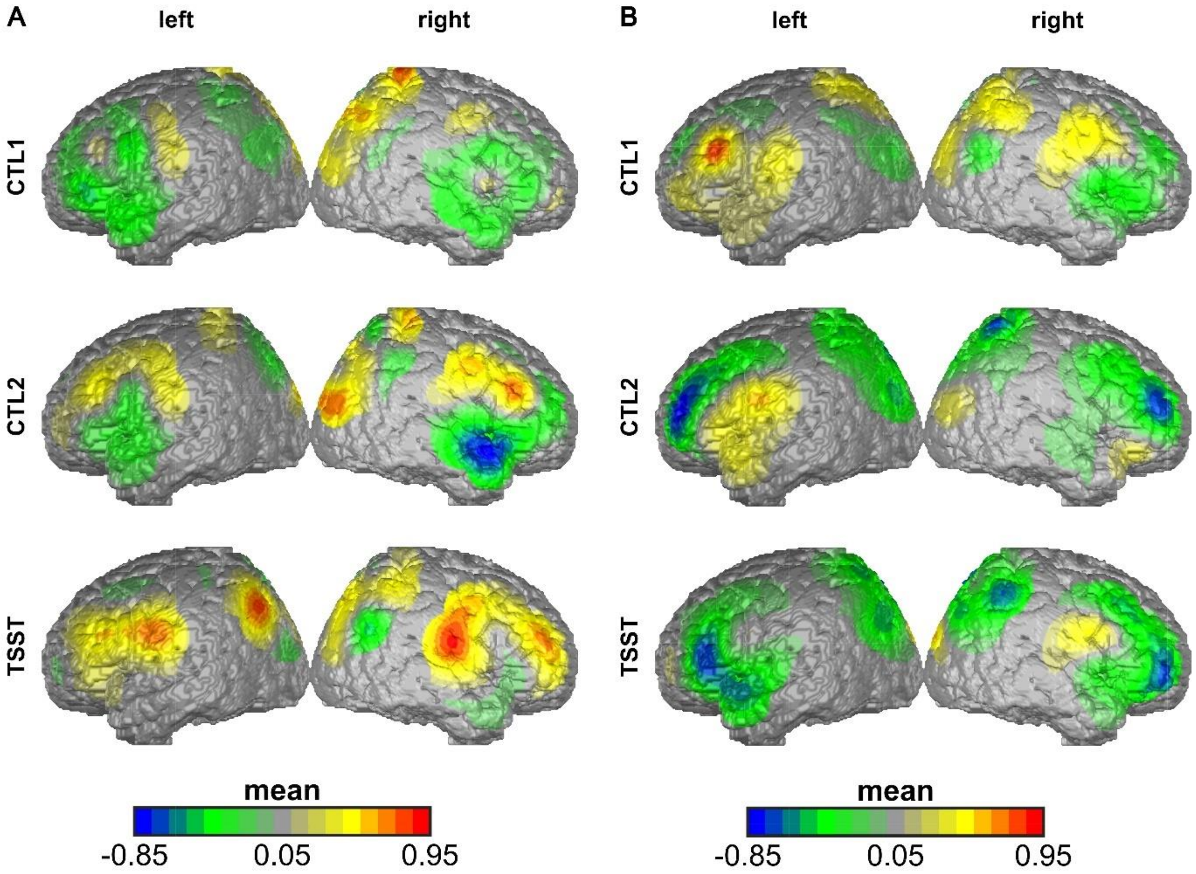

Figure S10.1. Standardized mean cortical oxygenation contrasts (active minus sham stimulation) of low ruminators having received A: cTBS or B: iTBS with the order of stimulation conditions active → sham; CTL1 = control task 1 (reading numbers), CTL2 = control task 2 (performing calculations without social stress); TSST = performing calculations under social stress. Warm colors indicate higher cortical oxygenation following the active stimulation compared to sham stimulation, cool colors indicate higher cortical oxygenation following sham stimulation compared to active stimulation.

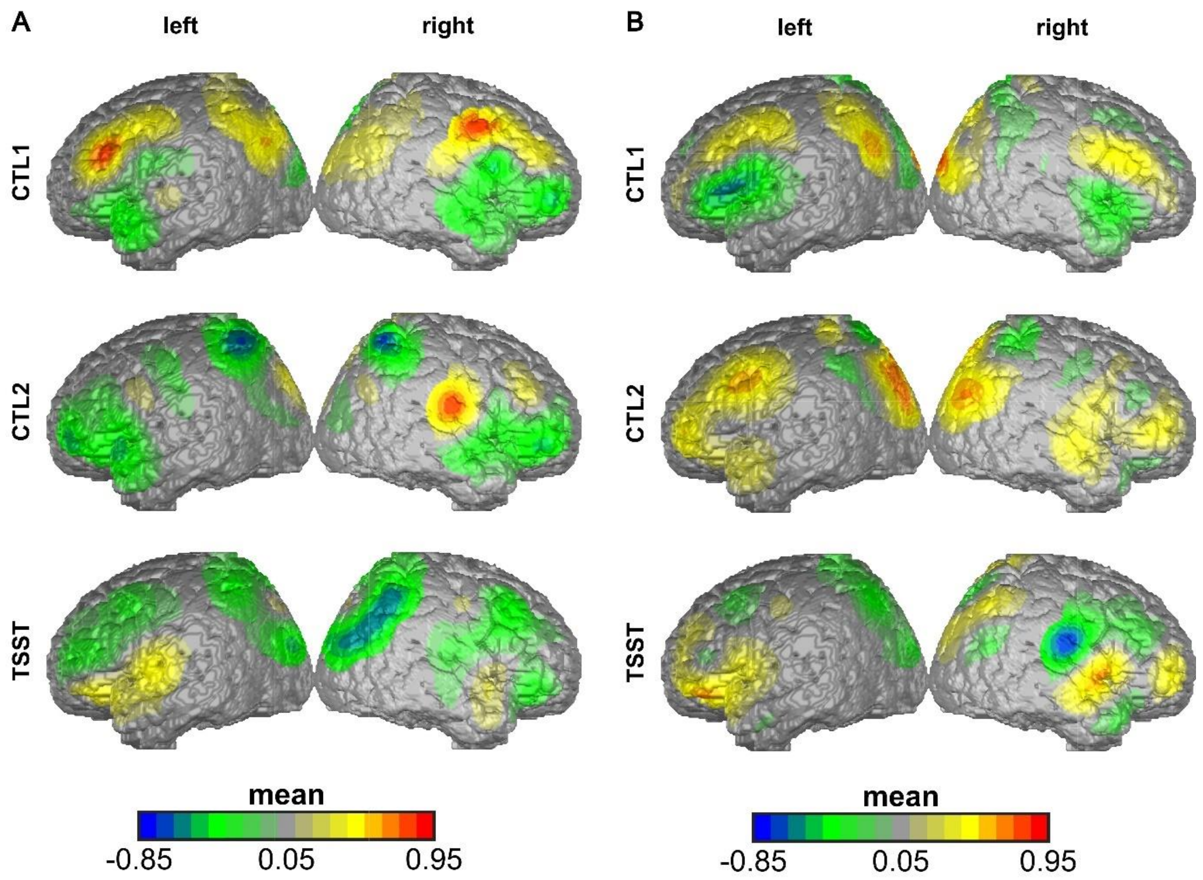

Figure S10.2. Standardized mean cortical oxygenation contrasts (active minus sham stimulation) of high ruminators having received A: cTBS or B: iTBS with the order of stimulation conditions active → sham; CTL1 = control task 1 (reading numbers), CTL2 = control task 2 (performing calculations without social stress); TSST = performing calculations under social stress. Warm colors indicate higher cortical oxygenation following the active stimulation compared to sham stimulation, cool colors indicate higher cortical oxygenation following sham stimulation compared to active stimulation.

Supplementary material S11: Time series of the hemodynamic responses during the arithmetic task of the TSST

Illustration of the z-standardized hemodynamic responses during the arithmetic task of the TSST in the Regions of Interest in low and high ruminators depending on the TBS-condition. A and B illustrate the raw data, while C and D illustrate the contrasts (cTBS minus sTBS and iTBS minus sTBS). The light shading marks the 40 s trial and the dark shading the 20 s pause to allow the hemodynamic response to recover. Shadings around the hemodynamic curves reflect standard errors of the mean. The baseline includes the 5 s before each trial; 0 s on the x-axis marks the beginning of the trial.

Time series of the first appointment:

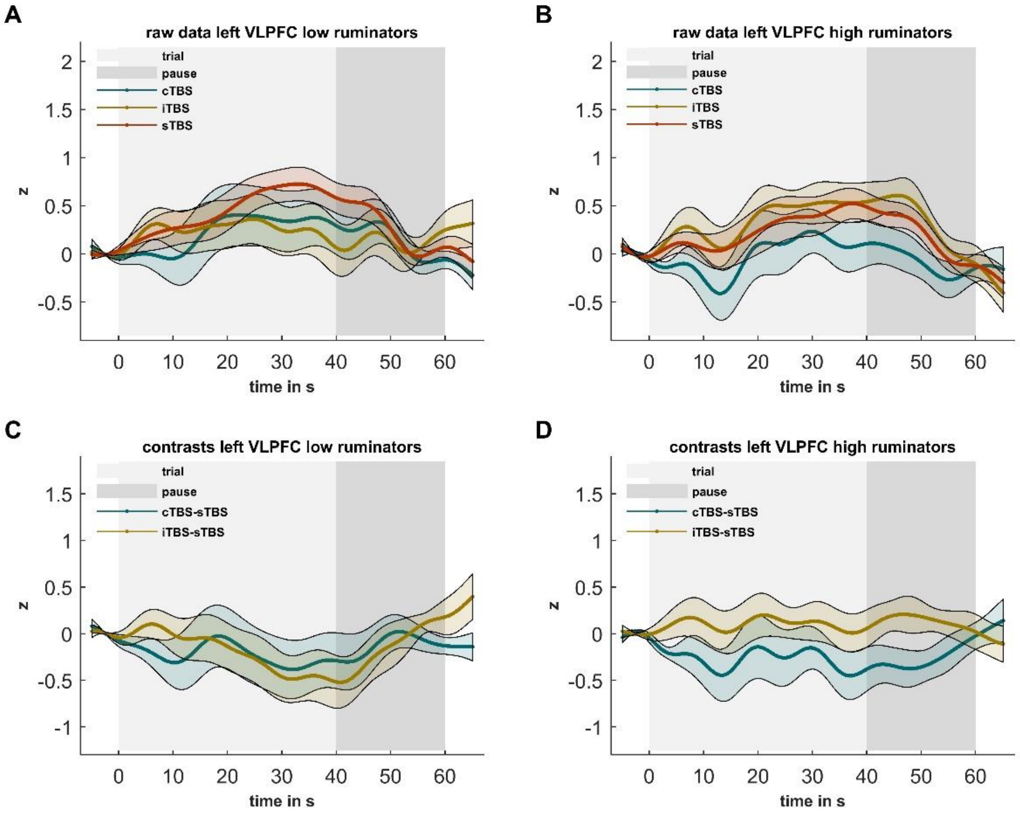

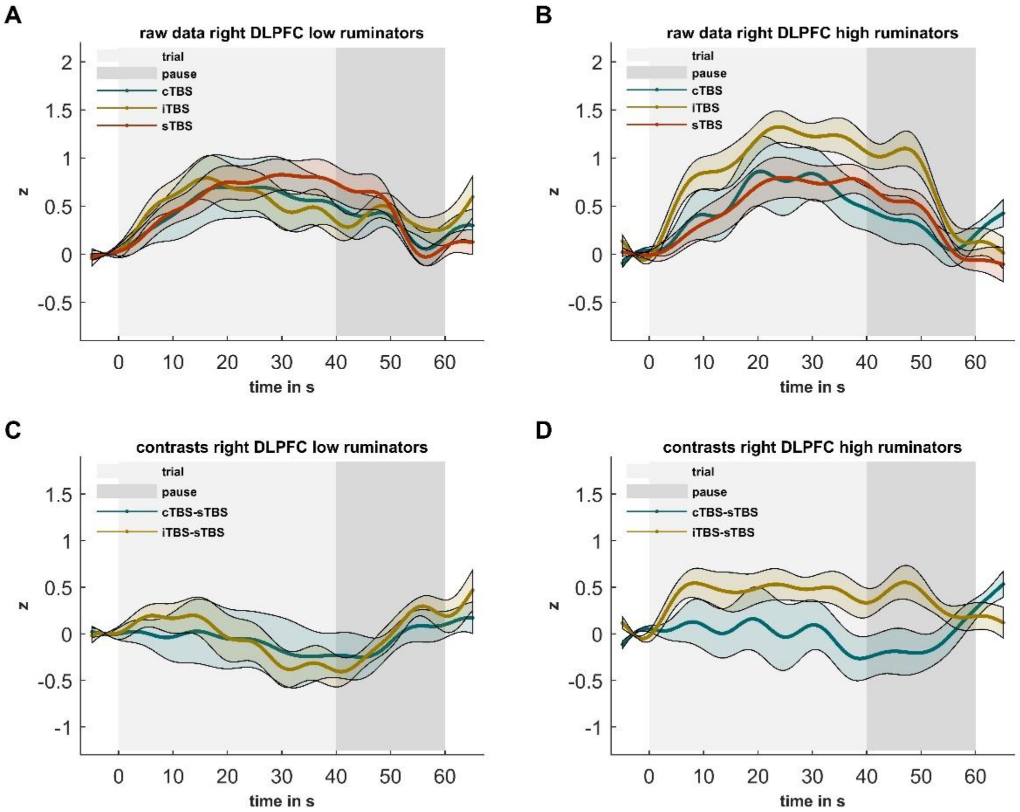
A
raw data right DLPFC low ruminators
B
raw data right DLPFC high ruminators
trial
pause
cTBS
iTBS
sTBS
z
time in s
C
contrasts right DLPFC low ruminators
D
contrasts right DLPFC high ruminators
cTBS-sTBS
iTBS-sTBS

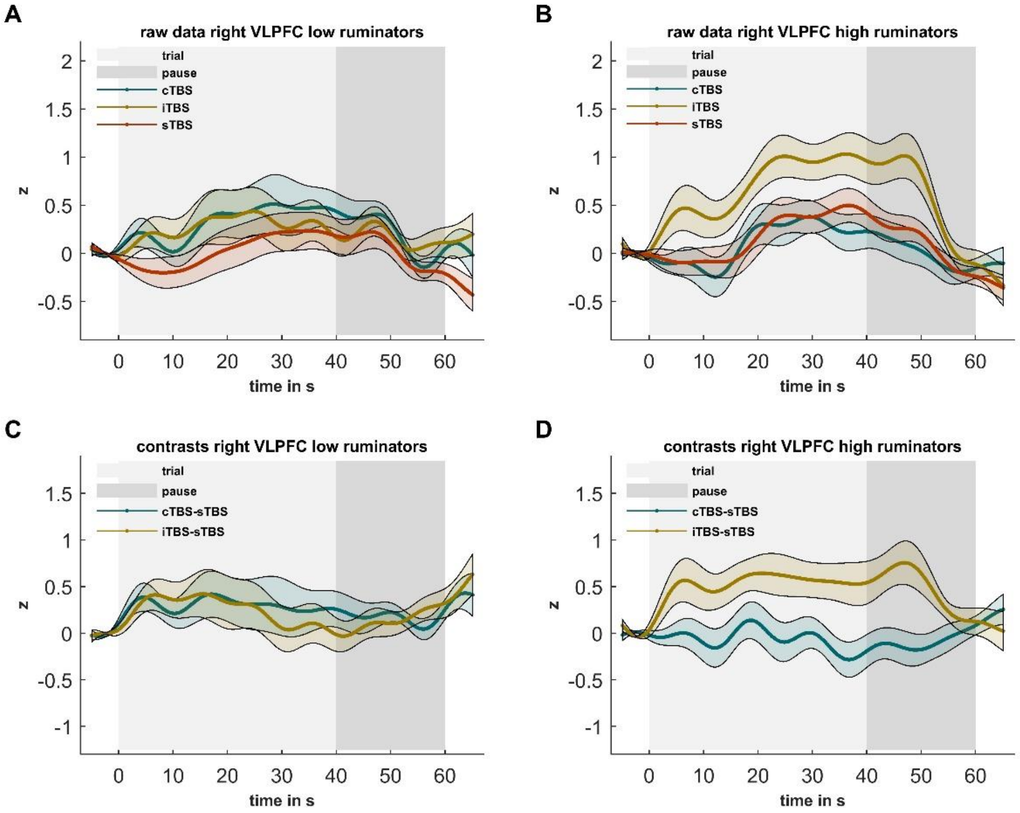
A
raw data right VLPFC low ruminators
B
raw data right VLPFC high ruminators
trial
pause
cTBS
iTBS
sTBS
z
time in s
C
contrasts right VLPFC low ruminators
D
contrasts right VLPFC high ruminators
cTBS-sTBS
iTBS-sTBS

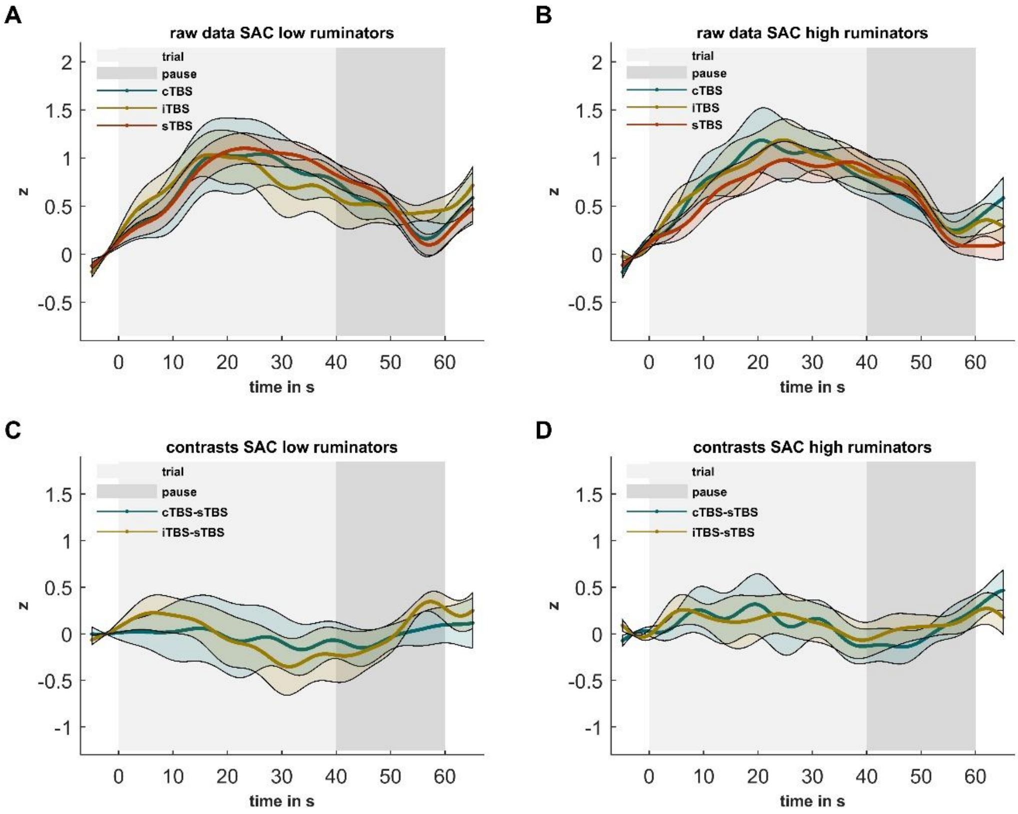

Time series of the second appointment:

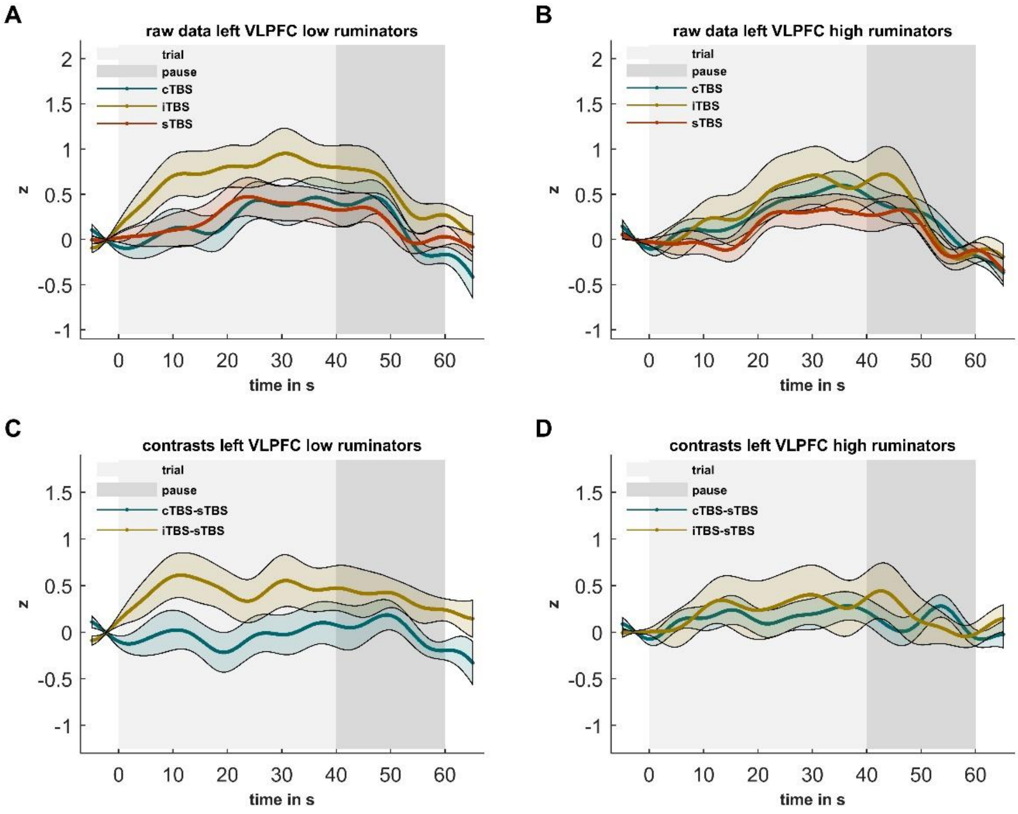

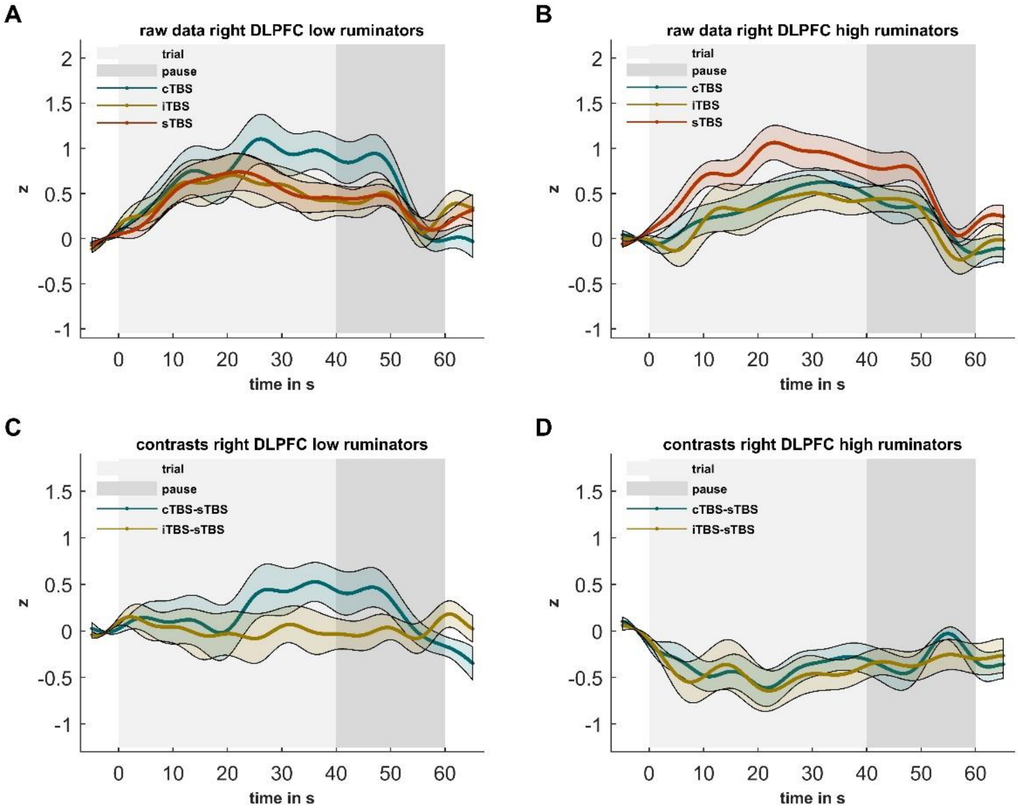
A
raw data right DLPFC low ruminators
B
raw data right DLPFC high ruminators
trial
pause
cTBS
iTBS
sTBS
C
contrasts right DLPFC low ruminators
D
contrasts right DLPFC high ruminators
cTBS-sTBS
iTBS-sTBS
z
time in s

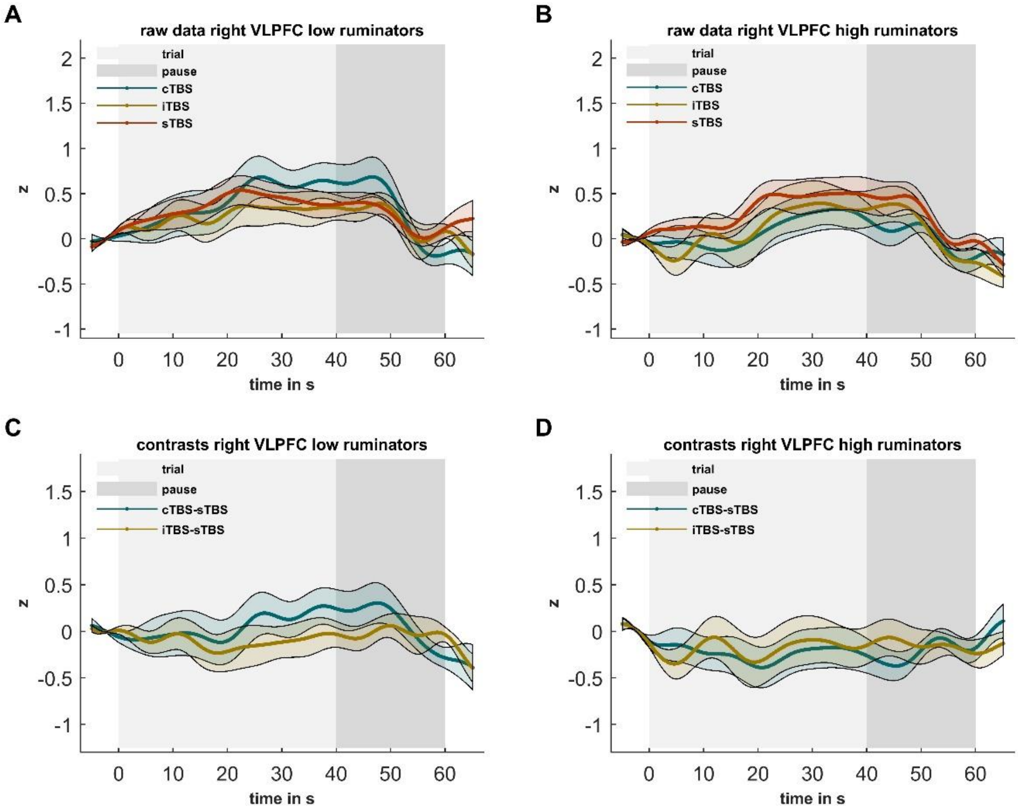
A
raw data right VLPFC low ruminators
B
raw data right VLPFC high ruminators
trial
pause
cTBS
iTBS
sTBS
C
contrasts right VLPFC low ruminators
D
contrasts right VLPFC high ruminators
cTBS-sTBS
iTBS-sTBS
z
time in s

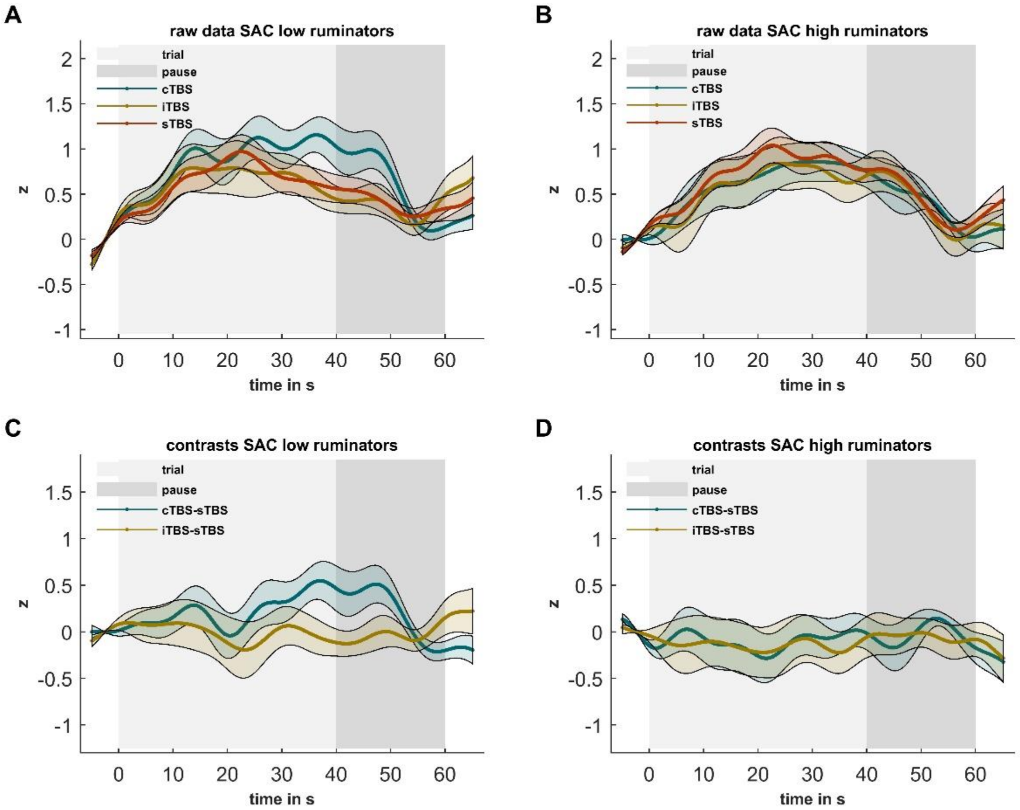
A
raw data SAC low ruminators
trial
pause
cTBS
iTBS
sTBS
z
time in s
B
raw data SAC high ruminators
trial
pause
cTBS
iTBS
sTBS
z
time in s
C
contrasts SAC low ruminators
trial
pause
cTBS-sTBS
iTBS-sTBS
z
time in s
D
contrasts SAC high ruminators
trial
pause
cTBS-sTBS
iTBS-sTBS
z
time in s

Supplementary material S12: Plots comparing state rumination induction in previous studies

Plots of state rumination as assessed in this study and in a previous study of our group: A = State rumination ratings dependent on RRS group in a study with similar set-up (Rosenbaum, Hilsendegen, et al., 2018) where low and high trait ruminator HC underwent the TSST but without a neurostimulation beforehand. The study consisted of only one appointment at the laboratory; B = State rumination ratings of the current study dependent on RRS group merged for both appointments; C = State rumination ratings of the current study dependent on RRS group only considering the first appointment; D = State rumination ratings of the current study dependent on RRS group only considering the second appointment. Please note that for both studies, the same cutoffs were used to determine RRS groups. RRS = Ruminative Response Scale
Please note that state rumination increases were in general low after the (TBS and) TSST (first appointment: $F(1, 85) = 16.188$, $p < .001$, $\eta_p^2 = .160$; second appointment: $F(1, 82) = 15.855$, $p < .001$, $\eta_p^2 = .162$) compared to Rosenbaum et al., 2018: $F(1, 44) = 19.832$, $p < .001$, $\eta_p^2 = .311$.

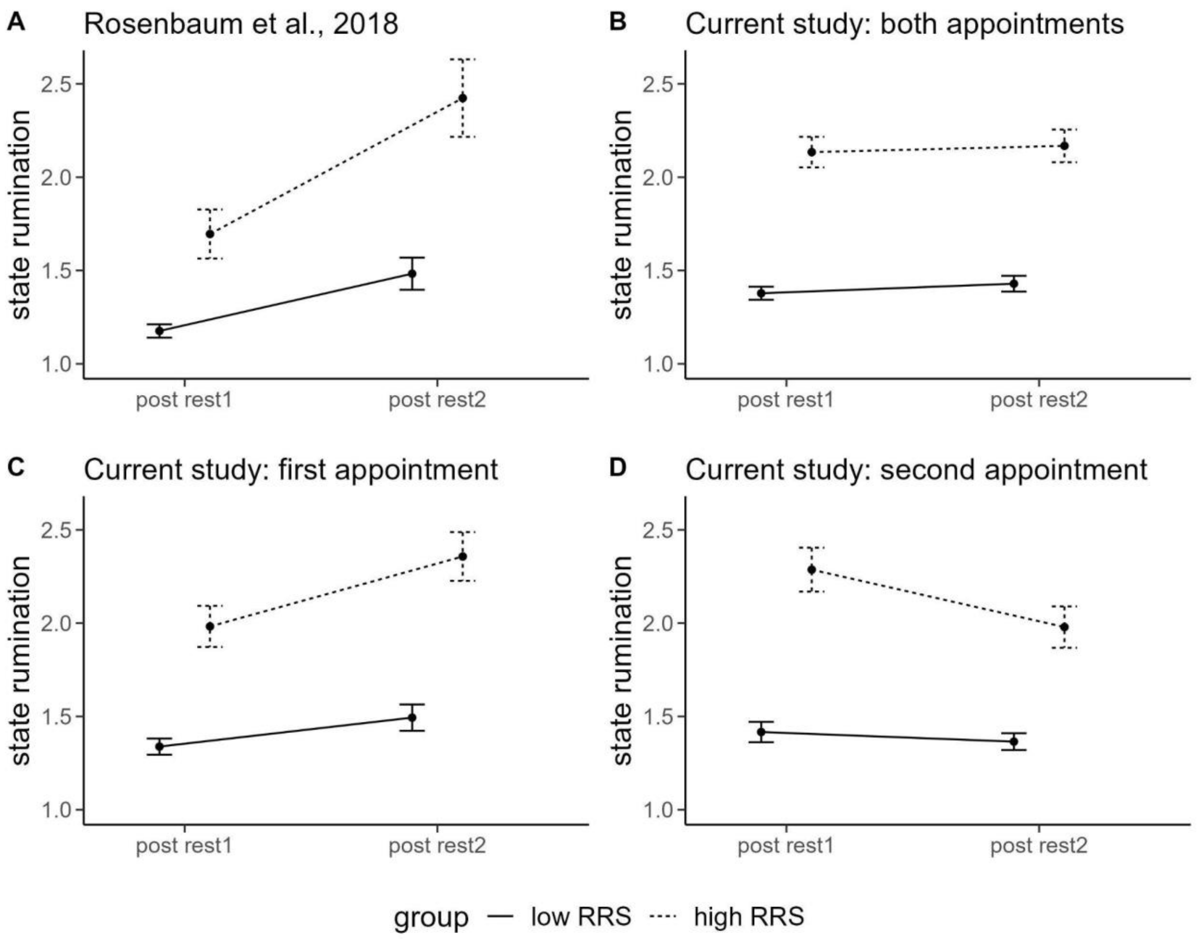

References of the supplementary material

De Witte, S., Baeken, C., Pulopulos, M. M., Josephy, H., Schiettecatte, J., Anckaert, E., De Raedt, R., & Vanderhasselt, M.-A. (2020). The effect of neurostimulation applied to the left dorsolateral prefrontal cortex on post-stress adaptation as a function of depressive brooding. *Progress in Neuro-Psychopharmacology and Biological Psychiatry*, *96*, 109687. https://doi.org/10.1016/j.pnpbp.2019.109687

Diaz, B. A., Van Der Sluis, S., Moens, S., Benjamins, J. S., Migliorati, F., Stoffers, D., Den Braber, A., Poil, S.-S., Hardstone, R., Van't Ent, D., Boomsma, D. I., De Geus, E., Mansvelder, H. D., Van Someren, E. J. W., & Linkenkaer-Hansen, K. (2013). The Amsterdam Resting-State Questionnaire reveals multiple phenotypes of resting-state cognition. *Frontiers in Human Neuroscience*, *7*. https://doi.org/10.3389/fnhum.2013.00446

Ehring, T., Zetsche, U., Weidacker, K., Wahl, K., Schönfeld, S., & Ehlers, A. (2011). The Perseverative Thinking Questionnaire (PTQ): Validation of a content-independent measure of repetitive negative thinking. *Journal of Behavior Therapy and Experimental Psychiatry*, *42*(2), 225–232. https://doi.org/10.1016/j.jbtep.2010.12.003

First, M., Williams, J., Karg, R., & Spitzer, R. (2015). *Structured Clinical Interview for DSM-5 Disorders, Clinical Trials Version (SCID-5-CT)*. American Psychiatric Association.

Nolen-Hoeksema, S., & Morrow, J. (1991). A prospective study of depression and posttraumatic stress symptoms after a natural disaster: The 1989 Loma Prieta earthquake. *Journal of Personality and Social Psychology*, *61*(1), 115–121. https://doi.org/10.1037/0022-3514.61.1.115

Rosenbaum, D., Hilsendegen, P., Thomas, M., Haeussinger, F. B., Metzger, F. G., Nuerk, H.-C., Fallgatter, A. J., Nieratschker, V., & Ehlis, A.-C. (2018). Cortical hemodynamic changes during the Trier Social Stress Test: An fNIRS study. *NeuroImage*, *171*, 107–115. https://doi.org/10.1016/j.neuroimage.2017.12.061

Rosenbaum, D., Int-Veen, I., Laicher, H., Torka, F., Kroczek, A., Rubel, J., Lawyer, G., Bürger, Z., Bihlmaier, I., Storchak, H., & others. (2021). Insights from a laboratory and naturalistic investigation on stress, rumination and frontal brain functioning in MDD: An fNIRS study. *Neurobiology of Stress*, 100344.

Rosenbaum, D., Int-Veen, I., Laicher, H., Woloszyn, L., Wiegand, A., Ladegast, S., Eßer, U., Kroczek, A., Sippel, D., Menkor, S., & others. (2024). Neural correlates of stress-reactive rumination in depression–the role of childhood trauma and social anxiety. *Neurobiology of Stress*, 100640.

Rosenbaum, D., Thomas, M., Hilsendegen, P., Metzger, F. G., Haeussinger, F. B., Nuerk, H.-C., Fallgatter, A. J., Nieratschker, V., & Ehlis, A.-C. (2018). Stress-related dysfunction of the right inferior frontal cortex in high ruminators: An fNIRS study. *NeuroImage: Clinical*, *18*, 510–517. https://doi.org/10.1016/j.nicl.2018.02.022

Thielscher, A., Antunes, A., & Saturnino, G. B. (2015). Field modeling for transcranial magnetic stimulation: A useful tool to understand the physiological effects of TMS? *2015 37th Annual International Conference of the IEEE Engineering in Medicine and Biology Society (EMBC)*, 222–225.
